# Medicinal Plants, Phytochemicals and Regulation of the NLRP3 Inflammasome in Inflammatory Bowel Diseases: A Comprehensive Review

**DOI:** 10.3390/metabo13060728

**Published:** 2023-06-06

**Authors:** Rosa Direito, Sandra Maria Barbalho, Maria Eduardo Figueira, Giulia Minniti, Gabriel Magno de Carvalho, Bárbara de Oliveira Zanuso, Ana Rita de Oliveira dos Santos, Natália de Góes Corrêa, Victória Dogani Rodrigues, Ricardo de Alvares Goulart, Elen Landgraf Guiguer, Adriano Cressoni Araújo, Henrique Bosso, Lucas Fornari Laurindo

**Affiliations:** 1 Laboratory of Systems Integration Pharmacology, Clinical & Regulatory Science, Research Institute for Medicines (iMed.ULisboa), Faculdade de Farmácia, Universidade de Lisboa, Av. Prof. Gama Pinto, 1649-003 Lisboa, Portugal; rdireito@ff.ulisboa.pt (R.D.); efigueira@ff.ulisboa.pt (M.E.F.); 2 Faculty of Pharmacy, Universidade de Lisboa, Av. Prof. Gama Pinto, 1649-003 Lisboa, Portugal; 3 Department of Biochemistry and Pharmacology, School of Medicine, University of Marília (UNIMAR), Avenida Hygino Muzzy Filho, 1001, Marília 17525-902, São Paulo, Brazil; smbarbalho@gmail.com (S.M.B.); gminniti27@gmail.com (G.M.); gabrielmagno60@gmail.com (G.M.d.C.); barbarazanuso98@gmail.com (B.d.O.Z.); anaritadeoliveirasantos@gmail.com (A.R.d.O.d.S.); nataliadegoescorrea@gmail.com (N.d.G.C.); rraartig@gmail.com (R.d.A.G.); elguiguer@gmail.com (E.L.G.); adrianocressoniaraujo@gmail.com (A.C.A.); 4 Postgraduate Program in Structural and Functional Interactions in Rehabilitation, University of Marília (UNIMAR), Avenida Hygino Muzzy Filho, 1001, Marília 17525-902, São Paulo, Brazil; 5 Department of Biochemistry and Nutrition, School of Food and Technology of Marília (FATEC), Avenida Castro Alves, 62, Marília 17500-000, São Paulo, Brazil; 6 Department of Biochemistry and Pharmacology, School of Medicine, Faculdade de Medicina de Marília (FAMEMA), Avenida Monte Carmelo, 800, Marília 17519-030, São Paulo, Brazil; vic8dr@gmail.com; 7 Medical Department, School of Medicine, Faculdade de Medicina de São José do Rio Preto (FAMERP), Avenida Brigadeiro Faria Lima, 5416, São José do Rio Preto 15090-000, São Paulo, Brazil; henriquebosso@gmail.com

**Keywords:** NLRP3, NLR Family Pyrin Domain Containing 3, inflammasome, inflammatory bowel disease, ulcerative colitis, cancer, medicinal plants, phytochemicals, Crohn’s disease, inflammation

## Abstract

Ongoing research explores the underlying causes of ulcerative colitis and Crohn’s disease. Many experts suggest that dysbiosis in the gut microbiota and genetic, immunological, and environmental factors play significant roles. The term “microbiota” pertains to the collective community of microorganisms, including bacteria, viruses, and fungi, that reside within the gastrointestinal tract, with a particular emphasis on the colon. When there is an imbalance or disruption in the composition of the gut microbiota, it is referred to as dysbiosis. Dysbiosis can trigger inflammation in the intestinal cells and disrupt the innate immune system, leading to oxidative stress, redox signaling, electrophilic stress, and inflammation. The Nod-like Receptor (NLR) Family Pyrin Domain Containing 3 (NLRP3) inflammasome, a key regulator found in immunological and epithelial cells, is crucial in inducing inflammatory diseases, promoting immune responses to the gut microbiota, and regulating the integrity of the intestinal epithelium. Its downstream effectors include caspase-1 and interleukin (IL)-1β. The present study investigated the therapeutic potential of 13 medicinal plants, such as *Litsea cubeba*, *Artemisia anomala*, *Piper nigrum*, *Morus macroura*, and *Agrimonia pilosa*, and 29 phytocompounds such as artemisitene, morroniside, protopine, ferulic acid, quercetin, picroside II, and hydroxytyrosol on in vitro and in vivo models of inflammatory bowel diseases (IBD), with a focus on their effects on the NLRP3 inflammasome. The observed effects of these treatments included reductions in IL-1β, tumor necrosis factor-alpha, IL-6, interferon-gamma, and caspase levels, and increased expression of antioxidant enzymes, IL-4, and IL-10, as well as regulation of gut microbiota. These effects could potentially provide substantial advantages in treating IBD with few or no adverse effects as caused by synthetic anti-inflammatory and immunomodulated drugs. However, additional research is necessary to validate these findings clinically and to develop effective treatments that can benefit individuals who suffer from these diseases.

## 1. Introduction

For thousands of years, plants have been known to be a source of direct health effects related to the treatment of common diseases [1,2]. Baradaran Rahimi et al. [3] have shown that *Boswellia serrata* could be a therapeutic agent against lipopolysaccharide-induced endotoxemia cardiac injury in H9c2 cells via anti-inflammatory and antioxidant effects. In turn, Pereira et al. [4] demonstrated that essential oils extracted from Tunisian flora plants such as rosemary (*Rosmarinus officinalis*), oregano (*Origanum vulgare*), prickly juniper (*Juniperus oxycedrus*), sage (*Salvia officinalis*), wormwood (*Artemisia herba-alba*), black cumin (*Nigella sativa*), geranium (*Pelargonium graveolens*), and coriander (*Coriandrum sativum*) could be valuable sources of anti-inflammatory agents capable of promoting beneficial health effects by lowering the levels of THP-1 macrophage inflammatory mediators that contribute to the development of various inflammatory diseases. Plant and plant-derived phytocompounds or phytochemicals have been considered due to their low costs, therapeutic application, and fewer side effects that promote an adequate therapeutic approach to various inflammatory and immunomodulated conditions. Although many synthetic drugs have been designed chemically, even more drugs have originated from medicinal plants. One example is the case of Inflammatory Bowel Diseases (IBDs), which are mainly treated nowadays with surgery and synthetic drugs that may not present effectiveness in many patients and can cause serious adverse effects [5,6].

Ulcerative Colitis (UC) and Crohn’s Disease (CD) are incurable, inflammatory gastrointestinal tract diseases that primarily affect the intestine. Still, they can cause signs and symptoms of systemic inflammation resulting in extraintestinal manifestations. Although CD and UC cause gastrointestinal symptoms, these two diseases express histopathological features differently and can be differentiated by physical examination and colonoscopy. CD and UC are remissive and recurrent, with a peak of onset between 20 and 30 years, affecting approximately 0.3% of Western populations. The treatment aims to induce and sustain periods of remission, thereby minimizing patients’ hospitalization durations and reducing the need for surgical interventions. If CD and UC are not correctly treated, their course may be related to severe damage to bowel homeostasis leading to profound disabilities and reduced quality of life and work capacity. Common symptoms include diarrhea, rectal bleeding, and abdominal pain [6,7].

**Figure 1:**
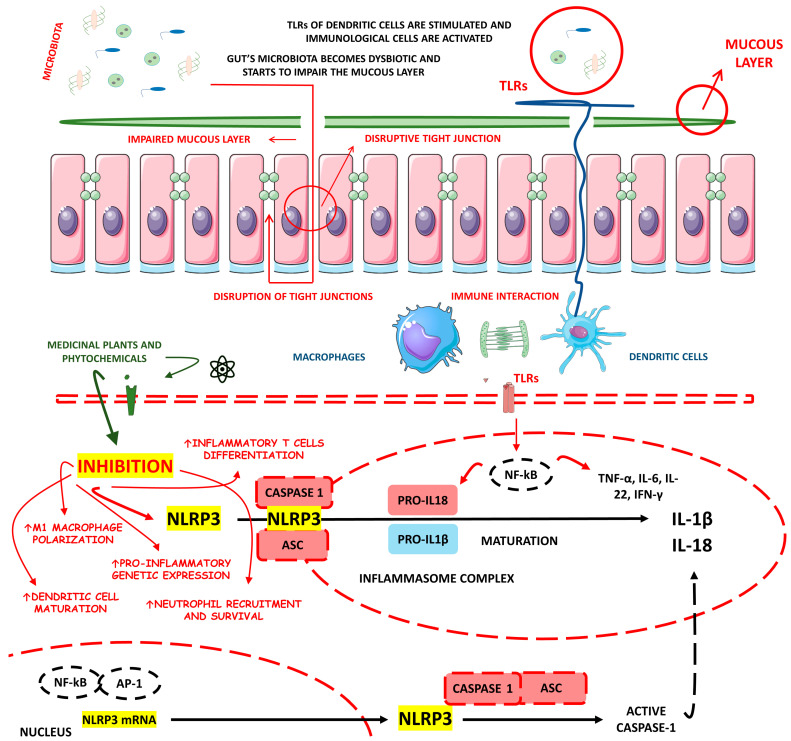
The potential impact of phytochemicals in mitigating NLRP3 pathway activation in the context of IBD. ASC, Apoptosis-Associated Speck-Like Protein Containing a CARD; IFN-γ, interferon gama; IL, Interleukin; mRNA, messenger RNA; NF-kB, nuclear factor kappa b; NLR, Nod-like Receptor; NLRP3, NLR Family Pyrin Domain Containing 3; TLRs, Toll-like receptors; TFN-α, tumor factor necrosis alfa.

The physiopathology of CD and UC is not yet completely understood. However, many authors highlight the potential of intestinal microbiota dysbiosis together with genetic, immunological, and environmental factors. Indeed, gut dysbiosis can trigger pro-inflammatory events throughout intestinal cells and dysregulation of the innate immune system. Moreover, oxidative stress, redox signaling, electrophilic stress, and massive inflammation can occur [8,9]. A master regulator of these processes is the Nod-like Receptor (NLR) Family Pyrin Domain Containing 3 (NLRP3) inflammasome, widely found in immunological and epithelial cells. NLRP3 possesses several roles during inflammatory disease induction and presents caspase-1 and interleukin (IL)-1β as their main downstream effectors. Recent studies advanced NLRP3 as a critical mediator of the immune response and a regulator of gut health by promoting immune responses to the bowel microbiota and controlling intestinal epithelium integrity [10,11,12]. Figure 1 illustrates the key pathways underlying the pathophysiology of IBD and highlights the disruptive impact of NLRP3 inflammasome activation on developing these diseases.

Many medicinal plants and phytochemicals have long been known as IBD regulators [6,13]. Accordingly, the plant kingdom may represent a paved road to constructing an effective treatment against CD and UC. For these reasons, the primary objective of this study was to conduct a comprehensive review of both in vitro and in vivo studies, focusing on medicinal plants, phytochemicals, and their regulatory effects on the NLRP3 inflammasome in the context of IBD.

## 2. Methodology for the Literature Search and Included Studies

### 2.1. Focal Question

The central question guiding the development of this review was: “What are the Effects of Medicinal Plants, Natural and Synthetized Phytochemicals as Regulators of the NLRP3 Inflammasome in Inflammatory Bowel Diseases”?

### 2.2. Language

This review exclusively incorporated studies that were written in English.

### 2.3. Databases

PubMed, Google Scholar, EMBASE, and COCHRANE databases were searched. The mesh terms employed in this study were “natural phytochemicals”, “synthesized phytochemicals”, “medicinal plants”, and “herbal medicine” in combination with “ulcerative colitis”, “Crohn’s dis-ease”, “inflammatory bowel diseases”, “NLR Family Pyrin Domain Containing 3”, and “NLRP3”. The implementation of mesh terms facilitated the search and identification of both in vivo and in vitro studies pertaining to the objectives of this review. No filters or restrictions were applied using the databases tools during the search and identification of the included in vivo and in vitro studies.

### 2.4. Study Selection

Due to the limited availability of clinical studies explicitly investigating the regulation of the NLRP3 inflammasome by medicinal plants and natural/synthesized phytochemicals in humans with IBD, this review exclusively included in vitro and in vivo studies in the final analyses. No specific inclusion or exclusion criteria were applied in selecting in vitro and in vivo studies for our review. All relevant studies pertaining to the aspects under investigation were included. Only full texts were considered.

### 2.5. Data Extraction

**Figure 2:**
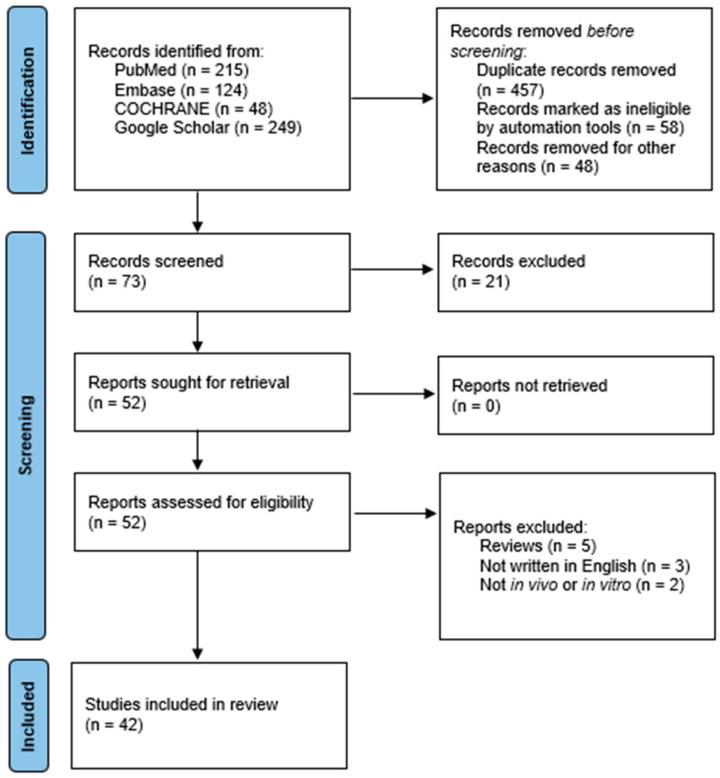
Flow diagram illustrating the literature search methodology of this review.

There was no time limitation on the search for in vivo and in vitro studies. The studies investigating the utilization of medicinal plants and natural and synthesized phytochemicals for regulating the NLRP3 inflammasome in IBD are listed in Table 1 and Table 2, respectively. Figure 2 illustrates the literature search.

### 2.6. Quality Assessment

The quality assessment was performed by two independent reviewers trained in utilizing the Scale for Assessment of Narrative Review Articles (SANRA). SANRA is a six-item scale developed by Baethge et al. [14] specifically designed to evaluate the quality of non-systematic reviews. The six items that compose the scale are “Justification of the article’s importance for the readership”, “Statement of concrete aims of formulation of questions”, “Description of the literature search”, “Referencing”, “Scientific reasoning”, and “Appropriate presentation of data”. The six items were rated on a scale from 0 (low standard) to 2 (high standard). To assist users in completing the scale, anchor definitions and examples have been developed to provide guidance and clarity. These guidelines aim to ensure consistent and accurate ratings of each item on the scale. A third independent reviewer resolved discrepancies between the two original reviewers.

**Table 1:** Studies regarding the use of medicinal plants with regulatory effects on NLRP3 inflammasome in inflammatory bowel diseases.

Medicinal Plants	In Vivo/In Vitro Model	Plant Parts Used or Extracts	Effective Doses/Concentrations	Related Clinical Features of IBD	Related Molecular Mechanisms in Regulation of NLRP3 in IBD	Reference
Xianglian Pill	DSS-induced C57BL/6 mice model of colitis in vivo	XLP is composed of *Coptis chinensis* Franch, *Evodia rutaecarpa*, and *Aucklandia lappa* Decne	1.35, 2.7, and 5.4 mg/kg/day orally for 7 days in vivo	↑DAI, ↑intestinal inflammation, ↓colon length, ↑histopathologic injury, ↑fecal occult blood, ↑diarrhea, ↑necrosis and ↑mucosal layer loss	↓NLRP3, ↓caspase-1, ↓GSDMD-N, ↓TLR4, ↓MyD88, ↓NF-κB, ↓p–NF–κB, ↓IL-1β, ↓TNF-α, ↓IL-18, and ↓MPO	[15]
*Litsea cubeba*	LPS and ATP-stimulated J774A.1 cells in vitro and DSS-induced C57BL/6 mice model of colitis in vivo	Leaves ethanolic extract	12.5, 25, or 50 μg/mL incubated for 0.5 h in vitro and 20, 40, and 80 mg/kg/day orally for 7 days in vivo	↑Inflammation in vitro and ↑fecal occult blood, ↑diarrhea, ↓body weight, ↓colon length and splenomegaly in vivo	↓IL-1β, ↓ASC, ↓caspase-1, ↓NLRP3, ↓ROS, ↓pyroptosis, ↓IL-6, and ↓mitochondrial ROS/damage in vitro and ↓IL-1β and ↓IL-6 in vivo	[16]
*Artemisia anomala*	LPS-stimulated BMDMs cells in vitro and DSS-induced C57BL/6 mice model of colitis in vivo	Whole plant ethanolic extract	1 μg/mL incubated for 40 min in vitro and 40 or 80 mg/kg/day orally in vivo	↑Inflammation in vitro and ↑DAI, ↑intestinal inflammation, ↑cellular infiltration ↓colon length, ↓body weight and ↑fecal occult blood in vivo	↓IL-1β, ↓NLRP3, ↓ASC, ↓TAK1-JNK, ↓caspase-1, ↓p65 nuclear, ↓IκBα, ↓NF-kB, ↓lysosomal disruption, ↓ROS, ↓mitochondrial damage, and ↓TNF-α in vitro and ↓IL-1β in vivo	[17]
*Schisandra chinensis* (Turcz.) Baill.	DSS-induced C57BL/6 mice model of colitis in vivo	Fruit ethanolic extract	593.78 and 1187.55 mg/kg/day orally for 14 days	↑DAI, ↑intestinal inflammation, ↓colon length, ↑intestinal mucosa damage, ↓body weight, ↑histopathologic injury	↑SOD, ↓MDA, ↓MPO, ↑ZO-1, ↓IL-1β, ↓TNF-α, ↓IL-18, ↓TLR4, ↓p-p65, ↓p-IκB-α, ↓TLR4/NF-κB signaling, and ↓NLRP3	[18]
Wu-Mei-Wan	DSS-induced C57BL/6 mice model of colitis in vivo	A mixture of *Coptidis* rhizoma, *Phellodendri chinensis* cortex, *Zingiberis* rhizoma recens, *Typhonii* rhizoma, *Zanthoxyli*, *Mume* fructus, and *Ginseng radix* rhizoma	Doses of 5.4, 2.7, and 1.35 g/mL were given orally by gavage, and each mouse was given 0.2 mL in vivo	↑Intestinal inflammation, ↑intestinal mucosa damage, ↑colon necrosis, ↑epithelial defects, ↓colon length, ↑infiltration of inflammatory cells, and ↑crypt injury	↓NLRP3, ↓Notch-1, ↓NF-κBp65, ↓p-NF-κBp65, ↓NLRP3, ↓IL-18, ↓IL-6, ↓IL-1β, ↓co-expression of Notch-1, ↓IL-18, ↓TNF-α, ↓macrophages infiltration, and ↓IRF5	[19]
Kui Jie Tong	DSS-induced mice model of colitis in vivo	A mixture of *Verbenae* herb, *Euphorbiae Humifusae* herb, *Arecae semen*, *Aurantii fructus* immaturus and *Angelicae sinensis* radix	10 mL/kg/day orally twice daily for 7 days	↑DAI, ↑intestinal inflammation, ↓colon length, ↓body weight, ↑mucosal injury, ↑crypt architecture loss, and hematochezia	↓NLRP3, ↓ASC, ↓caspase-1, ↓NEK7, ↓pyroptosis, ↓IL-1β, ↓IL-18, ↓IL-33, and ↓GSDMD	[20]
*Piper nigrum*	DSS-induced BALB/c mice model of colitis in vivo	Seed extract	50 and 100 mg/kg/day of *Viphyllin* for 14 days before DSS treatment	↑DAI, ↑intestinal inflammation, ↓colon length, ↓body weight, ↑mucosal injury, ↑crypt architecture loss, and ↓goblet cells/epithelial cells	↓TNF-α, ↓IL-1β, ↓NLRP3, ↑claudin-1, ↑occludin, ↓OS, ↑SOD, ↑CAT, ↑GSH, and ↓MDA	[21]
*Morus macroura* Miq.	AA-induced mice model of colitis in vivo	Whole fruit extract	100, 200, or 300 mg/kg/day orally given for 7 days before induction	↑DAI, ↑intestinal inflammation, ↓colon length, ↑mucosa injury, and ↓intestinal crypts	↑miRNA-223, ↓TNFα, ↓NFκB-p65, ↓caspase-1, ↓NLRP3, ↑SOD, ↑GSH, ↓MDA, ↓IL-1β, ↓TNF-α, ↓IL-18, ↓ROS, and ↓nitrate/nitrite	[22]
*Patrinia villosa*	TNBS-induced Sprague Dawley rat model of colitis in vivo	Dry leaf extract	21, 43, 64 g/kg/day orally for 14 days	↓Body weight, ↑diarrhea, ↓colon length, ↑ulceration, ↑rectal bleeding, ↑unformed feces, ↑blood feces, ↑DAI, ↑infiltration of inflammatory cells, ↓goblet cells, and ↓mucous epithelium,	↓IL-1β, ↓TNF-α, ↓IL-6, ↓NF-κB, ↓p-NF-κB, ↓NLRP3, and ↓caspase-1	[23]
*Ficus pandurata* Hance	DSS-induced C57BL/6 mice model of colitis in vivo	Stem, leaf, root, and whole plant extract	24 and 48 g/kg/day orally for 12 days orally	↓Body weight, ↑diarrhea, ↑rectal bleeding, ↓mucosal layer, ↓goblet cells, ↑muscular layer, ↑intestinal permeability, and ↑inflammatory cells infiltration.	↓TLR4 expression, ↓MyD88 expression, ↓NF-κB expression, ↓phospho-NF-κB expression, ↑T-SOD, ↑GSH-Px, ↓MDA, ↓Keap1, ↓NOX2, ↓p22-phox, ↑Nrf2, ↑HO1, and ↑NQO1	[24]
*Agrimonia pilosa*	DSS-induced C57BL/6 mice model of colitis in vivo	-	3 and 6 g/kg/day orally for 8 days	↓Body weight, ↓colon length, ↑neutrophil infiltration, ↑crypt loss, ↓goblet cell, and ↑submucosal edema	↓TNF-α, ↓IL-6, ↓IL-1β, ↓p65, ↓p-p65, ↓NLRP3, ↓ASC, ↓caspase-1, ↓NF-kB, and ↓NLRP3	[25]
*Vitis vinifera*	DSS-induced C57BL/6 mice model of colitis in vivo	Seed proanthocyanidin extract	50 mg/kg/day orally for 21 days	↓Body weight, ↓colon length, ↑DAI, ↑epithelial destruction, ↑crypt abscess, ↑goblet cell, ↑submucosal edema, and ↑inflammatory cells infiltration	↓TNF-α, ↓IL-1β, ↑IL-10, ↓TNF-α mRNA, ↓IL-1β mRNA, ↑IL-10 mRNA, ↓NLRP3 mRNA, ↓ASC mRNA, ↓caspase-1 mRNA, ↓MDA, ↑SOD, and ↑GSH	[26]
*Lycium ruthenicum* Murray	DSS-induced C57BL/6 mice model of colitis in vivo	Dry fruit extract	500 mg/kg/day orally for 14 days	↓Body weight, ↑DAI score, ↓colon length, ↓goblet cell, ↓crypt, ↑mucosa ulceration, ↑inflammatory cells infiltration, ↑necrosis, ↑ cell apoptosis, and ↑inflammatory infiltration	↓TNF-α, ↓IL-1β, ↓IL-6, ↓IL-17A, ↓IFN-γ, ↑IL-10, ↓TLR4, ↓p-IκB, ↓NF-κB p65, ↓p-p65, ↓c-Jun, ↓p-STAT3, ↓COX-2, ↓iNOS, ↓NO, ↓PGE2, ↓p38 phosphorylation, ↓ERK phosphorylation, ↓JNK phosphorylation, ↓NLRP3, ↓ASC, ↓caspase-1, ↓IL-1β, ↓ROS, ↓MDA, ↑CAT, ↑SOD, and ↑GSH	[27]

↑, increase; ↓, decrease; AA, acetic acid; ASC, apoptosis-associated speck-like protein containing a CARD; ATP, adenosine triphosphate; CAT, catalase; COX-2, cyclooxygenase-2; DAI, disease activity index; DSS, dextran sulfate sodium; ERK, extracellular signal-regulated kinases; GSDMD, gasdermin D; GSH, glutathione; HO1, heme oxygenase 1; IFNγ, interferon γ; IL, interleukin; iNOS, induced nitric oxide synthase; IkBα, IkappaB kinase α; IRF5, interferon regulatory factor 5; Keap1, kelch-like ECH-associated protein 1; LPS, lipopolysaccharide; MDA, malondialdehyde; miRNA, micro RNA; MPO, myeloperoxidase; mRNA, messenger RNA; MyD88, myeloid differentiation primary response 88; NEK7, NIMA related kinase 7; NFkB, factor nuclear kappa B; NLRP3, Nod-like receptor Family pyrin domain containing 3; NO, nitric oxide; NOX2, NADPH oxidase 2; NQO1, NADPH quinone dehydrogenase 1; Nrf2, nuclear factor erythroid 2 related factor 2; OS, oxidative stress; PGE2, prostaglandin E2; PPARγ, peroxisome proliferator activated receptor γ; RNA, ribonucleic acid; ROS, reactive oxygen species; SOD, superoxide dismutase; STAT3, signaling transducer and activator of transcription 3; TAK1-JNK, mitogen activated protein kinase 7 c-Jun N-terminal kinase; TLR4, Toll-like receptor 4; TNBS, 2,4,6-trinitrobenzene sulphonic acid; TNFα, tumor necrosis factor α; XLP, Xianglian pills; ZO-1, zonula occludens-1.

**Table 2:** List of natural and synthetized phytochemicals with regulatory effects on NLRP3 inflammasome in IBD.

Compounds/Phytochemicals	In Vivo/In Vitro Model	Effective Doses/ Concentrations	Related Clinical Features of IBD	Related Molecular Mechanisms in Regulation of NLRP3 in IBD	Reference
Artemisitene ** 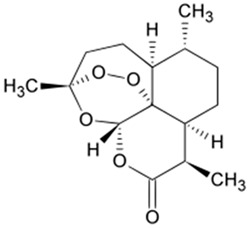 **	LPS, nigericin or ATP stimulated J774A.1 cells and LPS, nigericin, NLRC4 and AIM2 stimulated BMDMs cells in vitro and DSS-induced C57BL/6 mice model of colitis in vivo.	0.1, 1, or 5 μM incubated for 2 h or 24 h in vitro and 5, 15, or 45 mg/kg orally for 10 days starting from 3 days before colitis induction in vivo.	↑Inflammation and ↑OS in vitro and ↓colon length, ↑fecal occult blood, ↓body weight, ↑mucosal damages, ↑inflammatory infiltration, ↑goblet cell depletion, and ↑crypt architecture loss in vivo	↓IL-1β, ↓NLRP3-mediated IL-1β secretion, ↓NF-κB-dependent TNF-α activation, ↓pro-caspase-1 cleavage, ↓interaction between NLRP3 and ASC, ↓ASC oligomerization, ↓ASC specks, ↓NLRP3-ASC binding, ↓NLRC4, ↓AIM2 and ↓IL-6 in vitro and ↓IL-1β, ↓TNF-α and ↓IL-6 in vivo	[28]
Morroniside ** 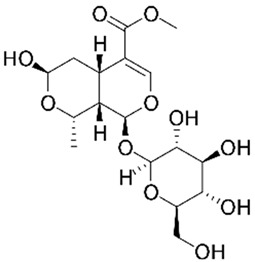 **	LPS-stimulated NCM460 cells in vitro	10, 30, 60, 100, or 200 µmol/L for 24 h in vitro	↑Inflammation and ↑cell injury in vitro	↓Bax, ↑Bcl-2 expression, ↓TNF-α, IL-1β and IL-6 expressions, ↑SOD and T-AOC expressions, ↓MDA and ↓MPO expressions, ↓NLRP3, ↓p-p65–p65 expression and ↓p-IκBα–IκBα expression	[29]
Protopine ** 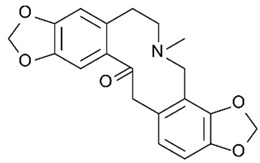 **	LPS-stimulated NCM460 cells in vitro	0, 5, 10, or 20 μM in vitro	↑Inflammation and ↑apoptosis and in vitro	↓Bax, ↑Bcl-2, ↓TNF-α, ↓IL-1β, ↓IL-6, ↑SOD, ↑T-AOC, ↓MDA, ↓MPO, ↓ROS, ↓intracellular Ca2+ concentration, ↑mitochondrial membrane potential, ↓NLRP3, ↓p-IκBα/IκBα and ↓p-P65/P65	[30]
Ferulic acid ** 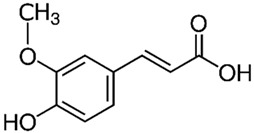 **	TNF-α-stimulated HIMECs cells in vitro and TNBS-induced Sprague-Dawley mice model of colitis in vivo	125, 250, or 500 μM incubated for 2 h in vitro and 10, 20, or 250 mg/kg/day rectally for 14 days in vivo	↓Cell proliferation and ↑cell apoptosis in vitro and ↓colon length, ↑inflammatory cells infiltration and ↑intestinal necrosis in vivo	↓IL-1β, ↓IL-6, ↓IL-12, ↓caspase-1, ↓caspase-3, ↓Bcl-2 and ↓TXNIP/NLRP3 in vitro and ↓IL-1β, ↓IL-6, ↓IL-12, ↓caspase-1, ↓caspase-3 and ↓TXNIP and ↓NLRP3 in vivo	[31]
Artemisinin analog SM934 ** 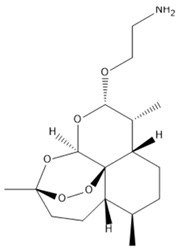 **	TNF-α-stimulated Caco-2 and HT-29 cells in vitro and TNBS-induced C57BL/6 mice model of colitis in vivo	10 μM incubated for 24 h and 10 μM incubated for 1 h in pre-treatment and 24 h or 72 h after treatment in vitro and 10 mg/kg/day orally from day 3 to day 7 in vivo	↑Inflammation and ↑OS in vitro and ↓body weight, ↓colon length, ↑epithelium erosion, ↑thickened intestinal wall, ↓crypt integrity, ↑mucosal injury, ↑inflammatory cells infiltration and ↑abnormal epithelial proliferation in vivo	↓c-Casp3, ↓NLRP3, ↑E-cadherin, ↑ZO-1, ↑occludin, ↓claudin-2, ↓ASC, ↓c-Casp1, ↓IL-18, ↓p-NF-κB, ↓p-p38, ↓p-ERK, ↓p-JNK, ↓GSDMD, ↓GSDMD-F and ↓GSDMD-N in vitro and ↓c-Casp3, ↓Bax/Bcl-2, ↓c-Casp9, ↓NLRP3, ↓ASC, ↓c-Casp1, ↓GSDMD, ↓L-18, ↓HMGB1, ↓NF κB, ↓ERK, ↓p38 and ↓JNK in vivo	[32]
Betaine ** 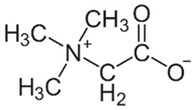 **	DSS-induced C57BL/6J mice model of colitis in vivo	600 mg/kg/day intraperitoneally for 7 days	↓Body weight, ↑DAI, ↑inflammatory cells infiltration, ↓goblet cells and ↓crypts integrity	↑Occludin, ↑ZO-1, ↓MDA, ↓MPO, ↓NOS-related enzymes, ↓COX2, ↑Nrf2, ↑CAT, ↑SOD, ↓NLRP3, ↓ASC, ↓c-Casp1 and ↓N-terminal GSDMD	[33]
N-Palmitoyl-D-Glucosamine	DNBS-induced C57BL/6J mice model of colitis in vivo	200 µL/day of 30 or 100 mg/kg of a PGA suspension orally from days 1 to 6	↑DAI, ↓colon length, ↑spleen weight, ↑intestinal neutrophil infiltration and ↓intestinal mucosa integrity	↑Occludin, ↑ZO-1, ↓TLR-4, ↓NLRP3, ↓iNOS, ↓IL-1β and PGE2 expressions	[34]
Moronic acid ** 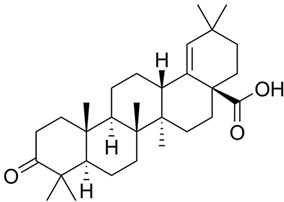 **	LPS/IFN-γ-stimulated intestinal macrophages in vitro and DSS-induced C57BL/6 mice model of colitis in vivo	10 or 20 μM incubated for 6 h in vitro and 5 or 10 mg/kg/day orally in vivo	↑Inflammatory phenotype in vitro and ↑inflammatory cells infiltration in vivo	↓TNF-α, ↓IL-1β, ↓IL-6, ↓ROS, ↓CD11, ↓NF-kB (P50), ↓NLRP3, ↓p-P50 and ↓M1 macrophage polarization in vitro and ↓ROS, ↓CD11, ↓TNF-α, ↓IL-1β, ↓IL-6, ↑ZO 1, ↓NLRP3 and ↓p-P50 in vivo	[35]
Munronoid I	LPS/ATP-stimulated mouse peritoneal macrophages and BMDMs cells in vitro and DSS-induced C57BL/6 mice model of colitis in vivo	0–50 mM incubated for 24 h in vitro and 10 mg/kg/day orally for 7 days in vivo	↑Cell injury and ↑formation of membrane pores in vitro and ↓body weight, ↑DAI, ↓colon length, ↑colon erythema, ↓epithelial cells integrity, ↑crypts distortion and ↑inflammatory cells infiltration in vivo	↓Caspase-1 p20, ↓IL-1β, ↓IL-18, ↓NLRP3 and ↓GSDMD p30 in vitro and ↓NLRP3, ↓cleaved caspase-1 (p20), ↓pyroptosis-related protein cleaved GSDMD (p30), ↓IL-6, ↓TNF-α, ↓IL-1β and ↓IL-18 in vivo	[36]
Sanguinarine ** 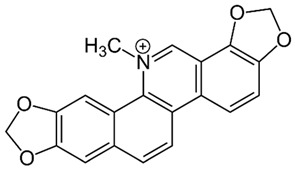 **	LPS-induced THP-1 cells in vitro and DSS-induced C57BL/6 mice model of colitis in vivo	0.25, 0.5, and 1.0 μM incubated for 1 h in vitro and 5 and 10 mg/kg twice daily orally for 7 days in vivo	↑Inflammation and ↑OS in vitro and ↓body weight, ↑DAI, ↓colon length, ↑spleen size, ↑irregular glands, ↑inflammatory cells infiltration, ↑TNF-α, ↑IFN-γ, ↑ IL-1β, ↑IL-6, ↑IL-13, ↑ IL-18, ↓IL-4, ↓IL-10 in vivo	↓NLRP3, ↓caspase-1, ↓IL-1β, ↓ROS and ↓IL-18 in vitro and ↓NLRP3, ↓caspase-1, ↓IL-1β, ↓TNF-α, ↓IFN-γ, ↓IL-1β, ↓IL-6, ↓IL-13, ↓IL-18, ↑IL-4 and ↑IL-10 in vivo	[37]
8-Oxypalmatine ** 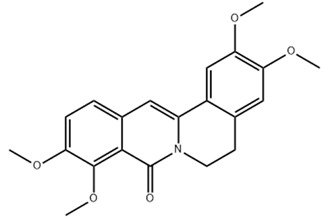 ** Oxidative metabolite	DSS-induced BALB/c mice model of acute colitis in vivo	12.5, 25, or 50 mg/kg/day of OPAL orally for 14 days	↑Diarrhea, ↓body weight, ↑rectal bleeding, colon shortening, ↑bloody stools, ↑inflammatory cell infiltration, ↑crypt damage, ↑epithelial cell destruction, and mucosal thickening	↓NLRP3, ↓ASC, and ↓Caspase-1 mRNA, ↓TNF-α, IL-1β, IFN-γ, IL-17A, IL-6 pro-inflammatory cytokines expression and secretion, ↑IL-10 expression and secretion, ↑SOD, GSH, CAT, GSH-Px antioxidant proteins expression and secretion, ↑Nrf2 and ↑HO-1 expression	[38]
Quercetin ** 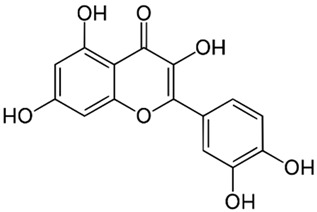 **	LPS-induced RIMVECs cells in vitro	300, 160, 80, 40, 20, 10, 5, 2.5, and 1.25 μM incubated for 12 h	↑Inflammation	↓TLR4, ↓NLRP3, ↓caspase-1, ↓GSDMD, ↓IL-1β, ↓IL-18, ↓IL-6, and ↓TNF-α	[39]
Picroside II ** 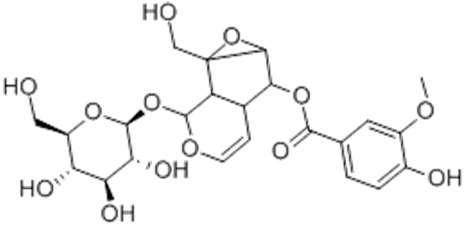 **	LPS-stimulated THP-1 treated cells in vitro and DSS-induced C57BL/6 mice model colitis in vivo	1, 5, 15, 30, 60, 100, or 150 μM incubated for 1 h in vitro and 5 or 10 mg/kg/day intraperitoneally for 7 days in vivo	↑Inflammation in vitro and ↑body weight loss, ↑tissue damage, ↑mucosal erosion and ulceration, and ↑inflammatory cell infiltration in vivo	↓NLRP3, ASC, and Caspase-1 proteins and ↓NF-κB signaling pathway in vitro, ↓NLRP3, ASC, and caspase-1 proteins in vivo and ↓TNF-a, ↓IL-6, and ↓IL-1β pro-inflammatory cytokines in vitro and in vivo	[40]
Hydroxytyrosol ** 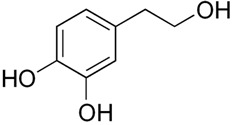 **	DSS-induced Kunming mice model of colitis in vivo	40 mg/kg/day orally for 14 days	↑Body weight loss, ↑colon shortening, ↑colonic morphologic damage, ↑desquamative epithelial cells in the lumen, ↑diffuse bleeding, ↓diversity levels of gut microbiota, and inflammatory cells infiltration	↓NLRP3, ASC, and Caspase-1 mRNA transcription, ↓NLRP3 inflammasome activation, ↓TNF-α, IL-1β, and IL-18 pro-inflammatory cytokines expression and secretion, ↓oxidative profiles (↓MPO, ↓MDA, ↑SOD, ↑GSH-Px, and ↑CAT enzymes expression), and ↓apoptotic-related proteins expression	[41]
SCLP	DSS-induced BALB/c mice model of acute colitis in vivo	250 and 500 mg/kg/day orally for 14 days	↑Body weight loss, ↓daily food intake, ↑colonic shortening, ↓goblet cells, ↑mucosal erosion, ↑cryptdamage and ↑infiltration of neutrophils and mononuclear cells	↓NLRP3, ASC, and Caspase-1 mRNA transcription and protein expression, ↓Gal-3 expression, ↓IL-6, TNF-α, and IL-1β pro-inflammatory cytokines expression and release, and ↓MPO expression	[42]
Bryodulcosigenin ** 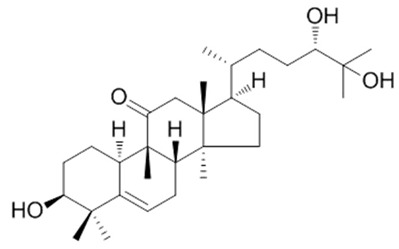 **	TNF-α-stimulated NCM460 treated cells in vitro and DSS-induced C57BL/6 mice model of colitis in vivo	10 μM incubated for 48 h in vitro and 10 mg/kg/day orally for 64 days in vivo	↑Inflammation in vitro and ↑body weight loss, ↑colonic shortening, ↑infiltration of inflammatory cells, ↑deformation of the crypt epithelium, and ↑necrosis of the crypt in the mucosal layer in vivo	↓NLRP3 inflammasome activation and ↓IL-1β and IL-17 mRNA expressions, ↓caspases cleavage and ↓Bax/Bcl-2 in vitro and ↓NLRP3 inflammasome activation, ↓IL-10, IL-1β, IL-17, and IL-6 mRNA expressions and ↑occludin and ↑ZO-1 expressions in vivo	[43]
Dioscin ** 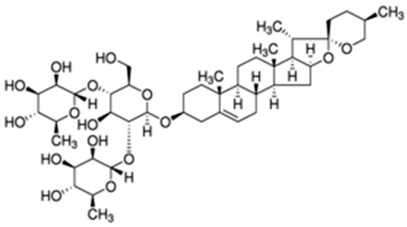 **	DSS-induced C57BL/6 mice model of acute colitis in vivo	40 mg/kg/day orally for 7 days	↑Colon shortening, ↑intestinal damage, ↑crypt shrinkage, and ↑inflammatory cells infiltration	↓NLRP3 inflammasome activation, ↓MAPKp38, ↓NF-κBp65 expression, ↓TNF-α and IL-1β pro-inflammatory expression, ↓M1 macrophage infiltration, ↓CD80 and ↑CD206 expressions and ↑IL-10 anti-inflammatory expression	[44]
Rosmarinic Acid ** 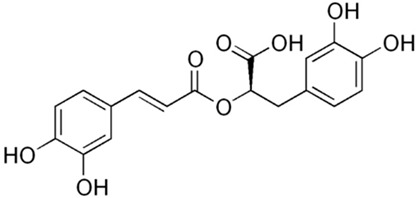 **	DSS-induced C57BL/6 mice model of colitis in vivo	5, 10, or 20 mg/kg/day orally for 7 days	↑Body weight loss, ↑colon shortening, ↑mucosal damage, ↑massive inflammatory infiltrate, ↑ulceration of the mucous epithelium, and ↓goblet cells	↓NLRP3 inflammasome activation, ↓NLRP3, ASC and caspase-1 expressions, ↓TNF-α, ↓MPO and ↓IL-1β expressions and ↑Nrf2 and ↑HO-1 expressions	[45]
Mogrol ** 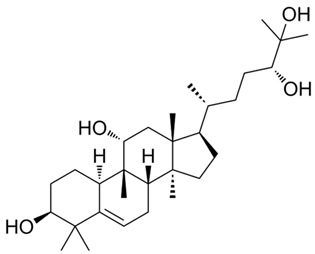 **	PMA-stimulated THP-1 treated cells and LPS-stimulated THP-M treated cells in vitro and DSS-induced C57BL/6 mice model of colitis in vivo	1 or 10 µM incubated for 24 h in vitro and 1 or 5 mg/kg/day orally for 7 days in vivo	↑Inflammation in vitro and ↑body weight loss, ↑colonic shortening, ↑crypt epithelium ↑distortion, and ↑inflammatory cells infiltration in vivo	↓NLRP3 inflammasome activation and ↓caspase-1 expression in vitro and ↓NLRP3 inflammasome activation, ↓NLRP3 mRNA, ↓NLRP3 activation, ↓IκBα degradation, ↓IL-1β and IL-17 pro-inflammatory cytokines expression, ↑IL-10 expression, and ↑occludin and ↑ZO-1 expressions in vivo	[46]
Sinapic Acid ** 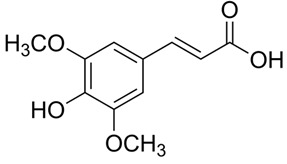 **	DSS-induced Kunming mice model of colitis in vivo	10 or 50 mg/kg/day orally for 7 days	↑Diarrhea, ↑gross bleeding, ↑body weight loss, ↑colon weight, ↑colon shortening, ↑disruption of the crypt epithelium, ↑mucosal damage and ↑inflammatory cells	↓NLRP3 inflammasome activation, ↓NLRP3, ASC and caspase-1 expressions, ↓TNF-α, IL-1β, IL-6, IL-8, IL-17α and ↓IFN-γ pro-inflammatory cytokines expression, ↑IL-4 and IL-10 anti-inflammatory cytokines expression, ↑SOD, GSH-Px, CAT and GSH expressions and ↓MDA and ↑claudin-1, occludin and ZO-1 proteins expression	[47]
Evodiamine ** 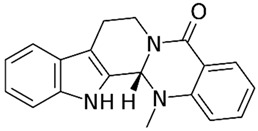 **	Human THP-1 cells stimulated by LPS in vitro and DSS-induced C57BL/6J mice model of colitis in vivo	10 μM incubated for 1 h in vitro and 20, 40, and 60 mg/kg/day orally for 10 days in vivo	↑Inflammation in vitro and ↓body weight, ↓colon length, ↑loss of tissue structure, ↑mucosal damage, ↑necrosis, ↑edema and ↑infiltration of inflammatory cells in vivo	↓IL-1β, ↓IL-18, ↓caspase1, ↓ASC and ASC oligomers, ↓P62 and ↑LC3 in vitro, ↓MPO, ↓IL-1β, ↓IL-18, ↓caspase1, ↓ASC, ↓p-P65NFκB, ↓p-IκB ↓P62 and ↑LC3 in vivo and ↓NLRP3 inflammasome assembly both in vivo and in vitro	[48]
Geniposide ** 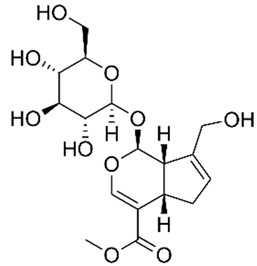 **	LPS-stimulated BMDM cells and RAW264.7 macrophages in vitro and DSS-induced C57BL/6J mice model of colitis in vivo	20, 50, and 100 μM incubated for 4 h in vitro and 25, 50 and 100 mg/kg/day orally for 7 days in vivo	↑Inflammation in vitro and ↑ulcer, ↓colon length and ↓histochemical score in vivo	↓IL-1β and ↓caspase-1 in vitro, ↓MPO, ↓IL-1β, ↓IL-17, ↓TNF-α, ↓IFN-γ, ↓caspase-1, ↓NOS2 mRNA, ↓Arg1 mRNA in vivo and ↓NLRP3 inflammasome pathway activation in both in vitro and in vivo	[49]
Chlorogenic Acid ** 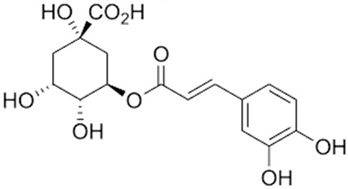 **	LPS/ATP-induced RAW264.7 macrophages in vitro and DSS-induced BALB/c mice model of colitis in vivo	0.5 µg/mL CGA incubated for 15 h in vitro and 20 and 40 mg/kg/day orally for 7 days in vivo	↑Inflammation in vitro and ↓body weight, ↓colon length, ↑loss of crypts, ↑bloody diarrhea and ↑inflammatory cells infiltration in vivo	↓IL-1β, ↓IL-18, ↓NLRP3, ↓ASC, ↓caspase1 p45, ↓caspase1 p20, ↓NF-κB and ↓MiR-155 in vitro and ↓IL-1β, ↓IL-18, ↓NLRP3, ↓ASC, ↓caspase1 p45, ↓caspase1 p20, ↑NF-κB and ↓MiR-155 in vivo	[50]
Resveratrol ** 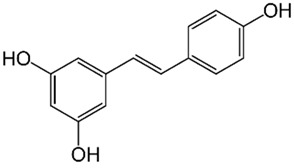 **	Radiation-induced C57/6 mice model of colitis in vivo	50, 100, and 200 mg/kg/day orally for 7 days before irradiation and then 14 days after irradiation.	↓Body weight, ↑tissue damage, ↑inflammatory cells infiltration and ↑mucosal edema	↓NLRP3, ↓Sirt1, ↓IL-1β and ↓TNF-α	[51]
Ginsenoside Rk3 ** 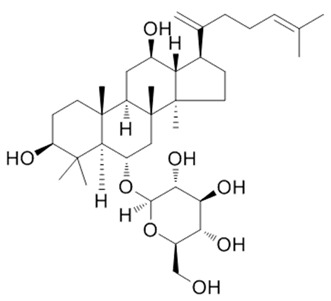 **	DSS-induced C57BL/6 mice model of colitis in vivo	20, 40, and 60 mg/kg/day orally for 14 days	↓Body weight, ↑DAI, ↓colon length, ↑inflammatory cells infiltration and ↓normal colon structure	↓NLRP3, ↓ASC, ↓caspase1, ↓MPO, ↓iNOS, ↓IL-1β, ↓TNF-α, ↓IL-6, ↑claudin 1, ↑occludin and ↑ZO-1	[52]
Physalin B ** 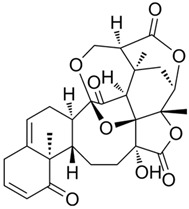 **	LPS-stimulated RAW 264.7 macrophages in vitro and DSS-induced BALB/c mice model of acute colitis in vivo	0.25, 0.5, 1.0, 2.0, 4.0, 8.0, and 16.0 µM incubated for 4 h in vitro and 10 and 20 mg/kg/day intraperitonially for 7 days in vivo	↑Inflammation in vitro and ↑DAI, ↓colon length, ↑inflammatory cells infiltration and ↓colon architecture in vivo	↓IL-1β, ↓IL-6 and ↓TNF-α in vitro and ↓NLRP3, ↓ASC, ↓IL-1β, ↓MPO, ↓TNF-α, ↓IL-6, ↓NF-κB activation cascade, ↓STAT3 and ↓β-arrestin 1 signaling pathway in vivo	[53]
Oroxindin ** 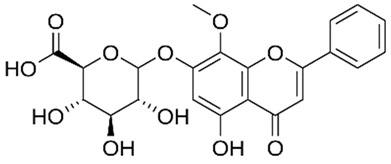 **	LPS-stimulated human THP-1 cells in vitro and DSS-induced C57BL/6 mice model of acute colitis in vivo	12.5, 25, and 50 μM incubated for 2 h in vitro and 12.5, 25, and 50 mg/kg/day via gastric intubation for 10 days in vivo	↑Inflammation in vitro and ↓body weight, ↓colon length, ↑spleen index, ↑white blood cell count, ↑colonic inflammation and ↑ulceration in vivo	↓NLRP3, ↓IL-1β, ↓caspase1 and ↓IL-18 in vtiro and ↓NLRP3, ↓IL-1β, ↓caspase1 and ↓IL-18 in vivo	[54]
Resveratrol Analog 2-Methoxyl-3,6-Dihydroxyl-IRA	Human colon cancer LS174T and Caco2 cells in vitro and DSS-induced C57BL/6 mice model of colitis in vivo	1 µM incubated for 24 h in vitro and 200 mg/kg/day orally for 9 days in vivo	↑Inflammation in vitro and ↓body weight, ↓colon length, ↑bleeding of anus, ↑tissue damage, ↑ulcerative areas and ↑epithelial necrosis in vivo	↓NLRP3, ↑Nrf2, ↑AKR1C, ↑NQO1, ↑GSH, ↓TNF-α and ↓IL-6 in vitro and ↓NLRP3, ↑Nrf2, ↑AKR1C, ↑NQO1, ↑GSH, ↓TNF-α and ↓IL-6 in vivo	[55]
Curcumin ** 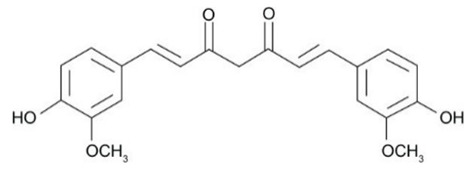 **	LPS-primed peritoneal macrophages in vitro and DSS-induced C57BL/6 mice model of colitis in vivo	0, 10, 25, and 50 μM incubated for 24 h in vitro and 100 mg/kg/day intraperitonially for 7 days in vivo	↑Inflammation in vitro and ↑DAI, ↓body weight, ↓colon length, ↑inflammatory cells infiltration and ↑tissue damage in vivo	↓IL-1β, ↑K^+^ efflux, ↓ROS production and ↓cathepsin B leakage in vitro and ↓NLRP3, ↓IL-1β, ↓ASC, ↑K^+^ efflux, ↓ROS production, ↓cathepsin B leakage, ↓caspase1, ↓MPO, ↓MCP-1 and ↓IL-6 in vivo	[56]
Dimethyl fumarate ** 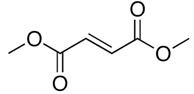 **	LPS-stimulated human THP-1 cells in vitro and DSS-induced C57BL/6 mice model of colitis in vivo	20 μM incubated for 12 h in vitro and 30 and 60 mg/kg/day orally for 10 days in vivo	↑Inflammation in vitro and ↓body weight, ↓colon length, ↑blood loss, stool consistency alterations, ↑DAI, ↑inflammatory cells infiltration, ↑tissue damage, ↑distortion of crypts and ↑loss of glandular epithelium in vivo	↓NLRP3, ↓IL-1β, ↓caspase1, ↓ASC, ↑Nrf2 and ↓ROS in vitro and ↓NLRP3, ↓IL-1β, ↓caspase1, ↓TNF-α, ↓IL-6, ↓MPO, ↓iNOS and ↑Nrf2 in vivo	[57]

↑, increase; ↓, decrease; AIM2, absent in melanoma 2; AKR1C, aldo-keto reductase family 1 member C1; AMP, adenosine monophosphate; AMPK, AMP-activated protein kinase; AOC, antioxidant capacity; ASC, apoptosis-associated speck-like protein containing a CARD; ATP, adenosine triphosphate; Bax, Bcl-2-associated X-protein; Bcl-2, B-cell lymphoma protein 2; BMDM, bone marrow derived macrophages; CAT, catalase; CD, cluster of differentiation; CGA, chlorogenic acid; COX-2, cyclooxygenase-2; DAI, disease activity index; DNBS, dinitrobenzene sulfonic acid; DSS, dextran sulfate sodium; ERK, extracellular signal-regulated kinases; GSDMD, gasdermin D; GSH, glutathione; HIMEC, human intestinal microvascular endothelial cells; HMGB1, high mobility group box 1; HO1, heme oxygenase 1; IFNγ, interferon γ; IL, interleukin; iNOS, induced nitric oxide synthase; IkBα, IkappaB kinase α; JNK, c-Jun N-terminal kinase; LC3, light chain 3; LPS, lipopolysaccharide; MAPK, mitogen-activated protein kinase; MCP-1, monocyte chemoattractant protein-1; MDA, malondialdehyde; MiR-155, micro RNA 155; MPO, myeloperoxidase; mRNA, messenger RNA; NFkB, factor nuclear kappa B; NLRC4, Nod-like receptor family caspase recruitment domain containing 4; NLRP3, Nod-like receptor family pyrin domain containing 3; NQO1, NADPH quinone dehydrogenase 1; NOS, nitric oxide synthase; Nrf2, nuclear factor erythroid 2 related factor 2; OPAL, 8-oxypalmatine; OS, oxidative stress; PGA, propylene glycol alginate; PMA, phorbol 12-myristate 13-acetate; PGE2, prostaglandin E2; RIMVEC, rat intestinal microvascular endothelial cells; RNA, ribonucleic acid; ROS, reactive oxygen species; SOD, superoxide dismutase; STAT3, signaling transducer and activator of transcription 3; TLR4, toll-like receptor 4; TNBS, 2,4,6-trinitrobenzene sulphonic acid; TNFα, tumor necrosis factor α; TXNIP, thioredoxin-interacting protein; ZO-1, zonula occludens-1.

## 3. Overview of the Included Pre-Clinical Studies

This manuscript comprised data from 13 different medicinal plants and 29 different phytocompounds used on in vitro and in vivo models of IBD. The included studies can be seen in Table 1 and Table 2, respectively. The in vivo studies used many different animal models of IBD such as dextran sulfate sodium (DSS)-induced mice model of colitis, 2,4,6-trinitrobenzene sulfonic acid (TNBS)-induced mice model of colitis, and acetic acid (AA)-induced mice model of colitis. Additionally, the in vitro models of IBD were lipopolysaccharides (LPS), nigericin and adenosine triphosphate (ATP)-stimulated J774A.1 cells, LPS-stimulated bone-marrow-derived macrophages (BMDMs) cells, tumor necrosis factor-α (TNF-α)-stimulated human intestinal microvascular endothelial cells (HIMEC), TNF-α-stimulated Caco-2 and HT-29 cells, LPS/Interferon-γ(IFN-γ)-stimulated intestinal macrophages, and LPS-induced THP-1 cells. The investigated medicinal plants and herbs formulations against IBD were Xianglian Pill, *Litsea cubeba*, *Artemisia anomala*, *Schisandra chinensis* (Turcz.) Baill., Wu-Mei-Wan, Kui Jie Tong, *Piper nigrum*, *Morus macroura* Miq., *Patrinia villosa*, *Ficus pandurata* Hance, *Agrimonia pilosa*, *Vitis vinifera*, and *Lycium ruthenicum* Murray. Additionally, the included phytocompounds used against IBD were artemisitene (sesquiterpene), morroniside (secoiridoid glycoside), protopine (benzylisoquinoline alkaloid), ferulic acid (hydroxycinnamic acid), artemisinin analog SM934, betaine (alpha amino acid), n-palmitoyl-d-glucosamine (monosaccharide-based glycolipid), moronic acid (pentacyclic triterpenoid), munronoid I, sanguinarine (benzophenanthridine alkaloid), 8-oxypalmatine (an oxidative metabolite), quercetin (flavonoid), picroside II, Hydroxytyrosol (catechol), SCLP (pectic polysaccharide purified from Smilax china), Bryodulcosigenin (cucurbitane-type triterpenoid), Dioscin (steroid saponin), rosmarinic acid (hydroxycinnamic acid), Mogrol (triterpenoid), sinapic acid (hydroxycinnamic acid), Evodiamine (alkaloid), geniposide (iridoid glycoside), chlorogenic acid (hydroxycinnamic acid), resveratrol (phytoalexin), ginsenoside RK3, physalin b (physalin), oroxindin (flavone), resveratrol analog 2-methoxyl-3,6-dihydroxyl-ira (synthetic), curcumin (curcuminoid), and dimethyl fumarate (FUMARIC ACID ESTER). Against IBD, the effects of medicinal plants and phytochemicals as regulators of the NLRP3 pathways were mainly decreases in the expression levels of major inflammasome regulators such as apoptosis-associated speck-like protein containing a CARD (ASC), NLRP3, and caspase-1.

The in vitro and in vivo models employed in the studies on IBD exhibit advantages and limitations that necessitate consideration. One commonly utilized model is the DSS-induced mice model of colitis, which offers the convenience of generating an acute, chronic, or relapsing model by simply adjusting the DSS administration concentration. Nevertheless, it should be noted that certain mouse strains can restrict or eliminate the inflammatory response induced by DSS in the colon. Furthermore, the TNBS-induced mice model of colitis also presents its advantages, including easy reproducibility, technical simplicity, and cost-effectiveness. However, it should be acknowledged that this model does not fully replicate the disease’s etiopathogenesis.

In Table 1, it is possible to observe that many different types of plant formulations and isolated parts of the plant were studied. The combination of *Coptis chinensis* Franch, *Evodia rutaecarpa*, and *Aucklandia lappa* Decne (XLP) [15] is efficient in down-regulating important inflammatory pathways as, among others, it can reduce the expression of NLRP3, IL-1β, il-18, and caspase-1. The other two herbal formulations (Wu-Mei-Wan [19] and Kui Jie Tong [20]) can act in the very same pathways. 

Leaf extracts were also investigated, and the results showed that leaf formulations obtained from *Litsea cubeba* [16] and *Patrinia villosa* [23] can promote similar results regarding the reduction in IL-1β, IL-6, TNF-α, caspase-1, and NLRP3.

Other authors evaluated the effects of the whole plant extracts obtained from *Artemisia anomala* [17] and *Ficus pandurata* [24] and found that these extracts can similarly reduce IL-1β, NLRP3, caspase-1, and NF-kB.

Fruit extracts also can diminish IL-1β and TNF-α levels and reduce the activity of NLRP3. These effects were observed with the fruit extract from *Shisandra chinensis* [18], *Morus macroura* [22], and *Lycium ruthenicum* Murray [27]. The same results can be observed using the Piper nigrum seed extract [21]. In one study, the authors investigated the effects of seed proanthocyanidin extract from *Vitis.* They found this isolated compound can reduce inflammation due to the reduction in pro-inflammatory biomarkers such as IL-1β, TNF-α, NLRP3 mRNA, and caspase-1 mRNA. 

The scenario of ROS decrease can usually be observed by the reduction in malondialdehyde and the upregulation of antioxidant enzymes such as SOD, CAT, and GSH. The reduction of ROS can also interfere with NLRP3 and directly improve IBD disease scores. These effects were observed by the use of the *Piper nigrum* seed, the fruit extracts obtained from *Shisandra chinensis* [18], *Morus macroura* [22], and *Lycium ruthenicum* Murray [27]; and extracts from the whole plant from *Artemisia anomala* [17] and *Ficus pandurata* [24]. Proanthocyanidin extract (from *Vitis* vinifera) can also reduce oxidative stress using the exact same mechanisms.

Several phytochemicals evaluated in Table 2 demonstrate potential in mitigating NLRP3 activation during IBD. These compounds exhibit reductions in inflammatory cytokines such as IL-1β and TNF-α and effects on NLRP3 activation-related effectors such as NLRP3, ASC, and pro-caspase 1. The phytochemicals showing both these beneficial effects include artemisitene, ferulic acid, artemisinin analog SM934, munronoid I, sanguinarine, 8-oxypalmatine, quercetin, picroside II, hydroxytyrosol, SCLP, rosmarinic acid, sinapic acid, evodiamine, chlorogenic acid, ginsenoside Rk3, physalin B, and dimethyl fumarate.

Furthermore, these same compounds also demonstrate the ability to reduce reactive oxygen species (ROS) levels. Phytochemicals such as protopine, moroninc acid, betaine, sanguinarine, hydroxytyrosol, curcumin, dimethyl fumarate, morroniside, 8-oxypalmatine, sinapic acid, and Resveratrol Analog 2-Methoxyl-3,6 Dihydroxyl-IRA contribute to the reduction of ROS, providing additional benefits in the context of NLRP3 activation during IBD.

## 4. IBD, NLRP3 Inflammasome, and Implications of Plant-Derived Interventions

### 4.1. Physiopathology of Ulcerative Colitis

UC is a chronic immune-mediated colon disease that is primarily an inflammatory disorder. In genetically susceptible individuals, it is hypothesized that exposure to environmental risk factors triggers an inappropriate immune response to the enteric commensal bacteria that compose the gut microbiota. Although only 8% to 14% of total patients affected by UC have a family history of IBD, first-degree relatives of individuals diagnosed with UC have four times the risk of developing the disease when in comparison with other patients. Additionally, monozygotic twins have concordance rates of 6% to 13% of developing UC [58,59]. Worldwide, UC prevalence is rising annually, ranging from 8.8 to 23.1 per 100,000 individuals in North American countries. UC is a disease that may occur during any age, but the peak of incidence in the 2nd to the 4th decade of life appears at the same incidences between men and women patients. The prevalence of UC has risen due to including phenotypes with lower mortality rates, younger ages of onset, and an absence of a potent cure. As complications, UC usually is accompanied by a recurrence of gastrointestinal infections, malignancies, and thromboembolic events [60,61,62,63]. 

Several different environmental factors serve as triggers for the development of UC. Before diagnosis, the onset usually has multiple gastrointestinal infections and exposure to non-selective non-steroidal anti-inflammatory drugs. These events might lead to profound changes in the bowel flora and trigger a chronic inflammatory process that, in genetically predisposed patients, could influence the occurrence of UC. On the other hand, appendicectomy has been used to treat UC and reduce the risk of the disease’s development [63,64,65,66].

Pathological changes in the intestinal epithelial barrier, gut microbiota, and immune system characterize UC. The disruption of enterocytes tight junctions and intestinal mucus layer covering the gut epithelial wall leads to increased permeability of the intestinal epithelium, allowing commensal bacteria to invade other layers of the bowel tissue. Innate immune cells such as dendritic cells and macrophages recognize the bacterial antigen of the commensals through Toll-like receptors (TLRs), switching from a tolerogenic to an active phenotype. These events stimulate the activation of the transcription of multiple pro-inflammatory genes and the production and secretion of various pro-inflammatory cytokines, including TNF-α, IL-12, IL-23, IL-1β, and IL-6 [64,67,68].

Once commensal bacterial antigens are processed, stimulated intestinal dendritic cells and macrophages present the antigens to naïve cluster of differentiation 4 (CD4) T cells. Consequently, these naïve cells differentiate into T helper (Th) 2 effector cells and produce the specific interleukin IL-4, which plays a role in the immune response against commensal bacteria. However, many other immune cells are involved in the pathogenesis of UC. Natural killer (NK) cells are the primary source of IL-13, linked to intestinal epithelial cell barrier disruption during the disease process. As the intestine becomes inflamed, it expresses more adhesive molecules to leukocytes, leading to the increased entry of T cells into the lamina propria of the bowel wall. Meanwhile, pro-inflammatory chemokines such as C-X-C Motif Chemokine Ligand (CXCL) 1 (CXCL1), CXCL3, and CXCL8 are upregulated in the intestine, perpetuating the inflammatory cycle of colitis [64,67,68,69].

The diagnosis of UC is based principally on clinical symptomatology and objective finding from the endoscopic examination. Additionally, histological examination can also be a priority. In UC, the inflammation generally starts in the rectum of the patients and extends to proximal areas uninterruptedly, involving part of or the entire colon. Clinically, the disease presents rectal bleeding, feces urgency, diarrhea, tenesmus, fever, and abdominal pain. The main endoscopic features are erythema, friability, granularity, loss of vascular patterns, ulcerations, spontaneous bleeding, and erosions. Histologically, the disease manifests distortion of crypt architecture, crypt abscesses, shortening of the crypts, erosions, ulcerations, lymphoid aggregates, mucin depletion, and lamina propria cellular infiltrate of plasma cells, lymphocytes, and eosinophils. Because diarrhea is a diagnostic criterion, it is fundamental to rule out infectious and non-infectious causes of diarrhea before a complete diagnosis. Depending on the involved affected segments of the colon, UC can be defined as proctitis, pancolitis, or left-sided colitis [63,64,68,70].

The treatment of UC involves mesalamine, immunosuppressive drugs, corticosteroids, and monoclonal antibodies against TNF-α [62,64,71]. The aims of a successful treatment consist of maintaining a steroid-free remission, mucosal healing, preventing surgery necessity as well as hospital admission, avoiding disability, and improving the quality of life for all patients [60,68,69]. Additionally, treatment must be tailored according to the disease extent (proctitis, pancolitis, or left-sided) and disease activity (mild, moderate, or severe). UC and CD patients often consider whether to withdraw treatment with their doctors, especially in cases of steroid-free remission. Previous research showed that up to 20 to 50% of patients at one year and 50 to 80% of patients beyond five years have a risk of relapse after stopping therapy, which is considered high. This data suggests that stopping treatment may not be a default strategy in fighting IBD. However, some patients may not experience relapse over a mid-term period, and others may benefit from a drug-free period before starting a new treatment cycle. Given the above, identifying the patients who can successfully withdraw from treatment must follow strict criteria. These may include the risk of relapse (related to factors such as mucosal healing and biomarkers), the consequences of a potential deterioration, the side effects and tolerance of the ongoing therapy, the patients’ priorities and preferences, and their disease’s costs. Ongoing research aims to provide a decisional algorithm that integrates these parameters and proposes to help patients and doctors make an appropriate decision for each of their cases [72,73,74].

### 4.2. Physiopathology of Crohn’s Disease

CD is a chronic inflammatory intestinal condition initially considered regional ileitis but is now recognized as affecting the entire intestine. During CD, the distal ileum is the most frequently affected area. Even with the improvements in alimentation, CD incidence is increasing worldwide, and patients experience periods of flares and remissions during the disease. Various risk factors influence CD pathophysiology, including environmental, immunological, genetic, and microbiota-related factors. Inflammation caused by inflammatory cells is a crucial driver of CD pathogenesis, making it a significant treatment target. Treatment aims to halt the inflammatory cascade by reducing the production of pro-inflammatory cytokines in the bowel [75,76,77,78].

As commented above, CD pathogenesis is based on the inflammation of the intestinal tissues, which is caused by an unrestrainable immunological response against the bacteria that compose the luminal microbiota of the human bowel. These bacteria present antigens that are recognized by antigen-presenting cells (APCs). Immune cells such as CD4 T lymphocytes, CD8 T lymphocytes, B-cells, NK cells, and CD14 monocytes start infiltrating the affected patients’ gut. Given the nature of the CD, it is known that immunological susceptibility in some innate immune response mechanisms in the defense against infections plays a critical role in the tolerance break of the immune system against intestinal bacteria. Reduced mucous production also helps this tolerance break insofar as it has been shown that variants of the Muc2 mucous-reduction genes are correlated with CD in many mouse models of the disease. People with altered interactions with the intestinal lumen commensal bacteria also have an increased risk of developing CD, such as in the case of the Galactoside alpha-(1,2)-fucosyltransferase 2 (FUT2) genetic variances, in which the patients encode a fucosyltransferase enzyme incapable of secreting antigens such as ABO [75,79,80].

The defects in the intestinal mucous barrier associated with CD involve primarily innate immunity. However, adaptive immunity relies on a Th1 lymphocytic immunological response mediated by pro-inflammatory cytokines such as TNF-α, IL-12, IL-23, and IL-34. Treg cells also contribute to the production of pro-inflammatory cytokines during CD development. The migration of Th1 lymphocytes and Treg cells to the affected sites is facilitated by interactions with integrins (such as the receptor for α4β4 integrin) and adhesion molecules (such as leucocyte MAcCAM-1), which are mediated by various chemokines and metalloproteinases. Matrix metalloproteinases (MMP-1 and MMP-3), produced through the stimulation of leukocyte proteins such as CD44 and CD26, are among the factors that contribute to the inflammatory response [75,77,81].

Although other T cell subsets than Th1 can emerge during CD. Th17 cells are relevant examples, whereas the most prominent involved pro-inflammatory cytokines are the TNF-α, IL-12, and IL-23. As aforementioned, the Th2 response is antagonistic to CD, because Th2 cells are characteristic of UC. Apart from those most common pro-inflammatory cytokines, IL-34 also plays a critical role during CD development and has been associated with areas of very active inflammation due to its power to induce the release of TNF-α and IL-6 through extracellular signal-regulated kinases (ERK)-mediated mechanisms and Chemokine (C-C motif) ligand (CCL) 20 (CCL20) through the interaction with its Macrophage colony-stimulating factor 1 (M-CSFR1) receptor. CD inflammation is typically transmural and, on the pathologists’ examinations, granulomas with a discontinuous distribution along the longitudinal axis are frequently found, leading to irreversible tissue damage due to intestinal stenosis and fistulas, inflammatory masses, or even intra-abdominal abscesses derived from possible concomitant infections [75,82,83].

The immune network that emerges in CD derives from the microbial-associated molecular pattern (MAMP) recognition by the leukocytes involved in the defense of the intestinal mucosa. Toll-like receptors sense these molecular bacterial patterns on immune cells, contributing to the disease’s chronic inflammation [75,83].

During the disease, common symptoms include diarrhea, fever, weight loss, abdominal pain, rectal bleeding, and fatigue. Physical examination is fundamental to identify the disease clinically, insofar as the anorectal examination. The search for extraintestinal manifestations and complications helps distinguish between other IBD patients. Fecal calprotectin can also be an alternative to diagnose CD in adults and children. However, cross-sectional endoscopy imagining is mandatory to confirm the diagnosis and measure the extent of the disease [76]. Despite the adverse effects, corticosteroids are still the choice to treat symptoms and flares. Biological medicaments and immunomodulators induce and maintain remissions [75,76,78,84].

**Figure 3:**
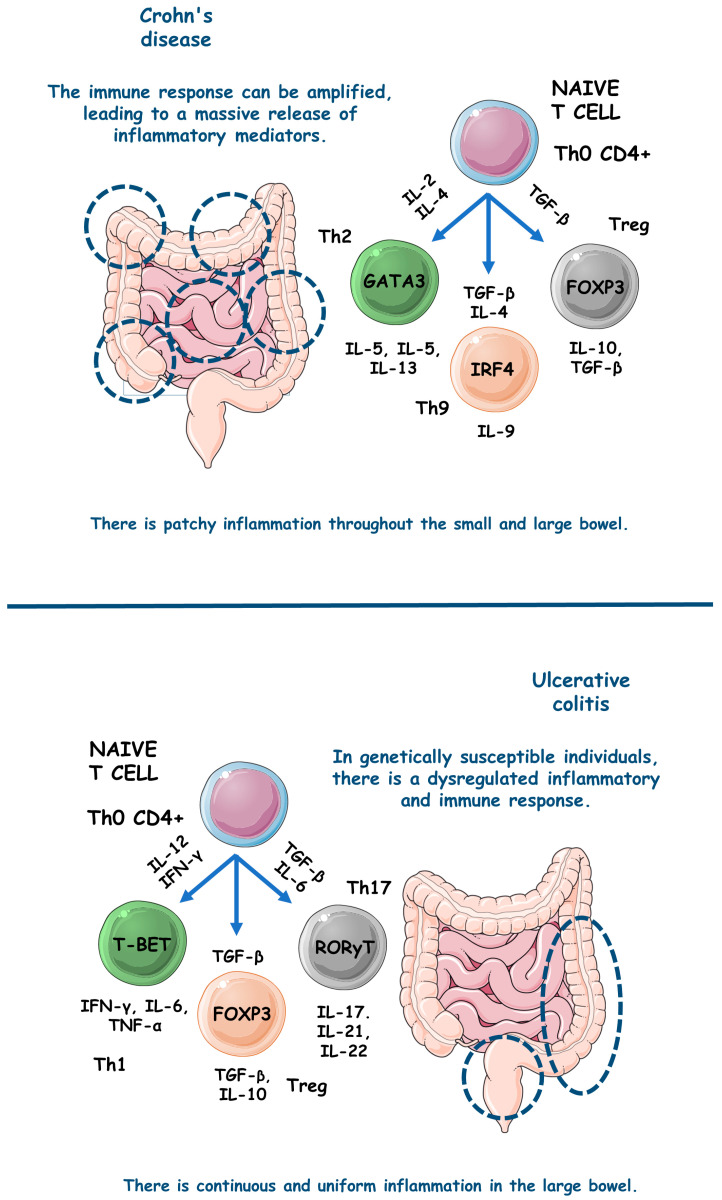
The main pathophysiological steps involved in the occurrence of UC and CD. ↑, increase; FOXP3, forkhead box P3; GATA3, GATA (Erythroid transcription factor) Binding Protein 3; IFN-γ, interferon gama; IL, interleukin; IRF4, Interferon regulatory factor 4; T-BET, T-box transcription factor TBX21; TGF-β, transforming growth factor beta; Th, T helper; TNF-α, tumor necrosis factor alfa; Treg, T regulatory cell.

Figure 3 comparatively exemplifies the pathophysiological steps involved in the occurrence of UC and CD.

### 4.3. NLRP3 Inflammasome: Implications for IBD

Inflammasomes are cytoplasmic multimeric complexes of proteins that not only mediate IL-1β and IL-18 activation but also induce pyroptosis. Primarily, inflammasomes are mainly responsible for initiating and sustaining the innate immunological response against many kinds of stressors (endogenous and exogenous). NLRP3 is unique among all NLR families of inflammasome receptors and is the only known member that seems to be indirectly activated through pathogenic and sterile pro-inflammatory signals. In the pathogenic field, bacterial and viral pathogen-associated molecular patterns (PAMPs) such as nucleic acids and LPS and other bacterial toxins such as nigericin and gramicidin activate the NLRP3 domain. In the sterile area, damage associated molecular patterns (DAMPs), reactive oxygen species (ROS), extracellular presence of ATP, potassium efflux, and various metabolic crystals can also activate the NLRP3 domain. NLRP3 depends on a sensor (NLRP3), an adaptor (ASC or PYARD), and an effector (caspase-1). Theoretically, priming and activation steps are necessary to activate NLRP3. In the priming step, three components (NLRP3, pro-IL-1-β, and caspase-1) must be upregulated, and PAMPs and DAMPs trigger this upregulation by stimulating pattern recognition receptors (PRRs) and cytokine receptors, including TLRs and IL-1R, respectively. Furthermore, many stimuli activate the proper NLRP3 regulator during the activation step. The incentives can be endogenous, such as DAMPs, or exogenous, such as PAMPs, K^+^, Cl^−^ ions efflux, or the flux of Ca^2+^ [85,86,87].

NLRs comprise an N-terminal domain called an effector, a C-terminal leucine-rich repeats (LRR), and a central nucleotide-binding domain (NBD/NOD/NACHT). The subdomains NACHT and LRR are the only ones conserved in all NLRs except NLRP10, and the N-terminal effector domain can vary. Due to N-terminal variance, NLRs can interact with various partners and recruit many integrators. The activation of NLRP3 inflammasome is evidenced during the pathogenesis of many different inflammatory and immunomodulated diseases such as diabetes, IBD, and atherosclerosis. However, to cause illness, NLRP3 must be overactivated. The most studied inflammatory pathway NLRs activate is NF-kB, but they also conduct *mitogen-activated protein kinase* (MAPK) signaling, antigen presentation, assembly of cytosolic signals transduction complexes, and embryonic development [88,89]. Thus, NLRP3 should be tightly regulated to prevent unwanted disease processes and body damage through excessive inflammation.

After sensing dangerous signals, which presumably occurred by the LRR domain of the NLRP3 complex, NLRP3 monomers start to induce oligomerization and interact with pyrin domains called PYD of ASC via homophilic interactions. Then, ASC, as an adaptor, becomes a recruiter of cysteine protease pro-caspase-1 through a caspase recruitment domain (CARD). As a result, the autocatalysis and further activation of caspase-1 lead to the induction of pro-inflammatory cytokines IL-1β and IL-18 production and pyroptosis. For the proximity-induced activation of NLRs inflammasomes such as NLRP3, recent structural studies revealed that two successive and interconnected steps in nucleation-induced and “prion-like” polymerization are necessary. These are the NLRP3 nucleation of the PYD filaments of ASC and pro-caspase-1 cluster within star-like fibers of ASC [88,90].

NLRP3 has also been observed to interact with nucleotide-binding oligomerization domain 2 (NOD2) in a CARD-dependent manner. This interaction plays a distinct role in processing the major NLRP3-associated pro-inflammatory cytokine IL-1β. Differently from other cytokines of the IL-1 family that can suppress inflammation, IL-1β stimulates it. Indeed, IL-1β is crucial in mediating pro-inflammatory responses in various tissues among multiple systemic inflammatory diseases and is associated highly with leukocytosis and elevated acute phase proteins [88,90].

Etiologically and as previously mentioned, IBD occurrence remains unclear. However, aberrant immunological responses against commensal intestinal bacteria are widely thought to underline IBD. A recent investigation found that IL-1β, not IL-18, is the most related to NLRP3 downstream during IBD and is also an essential effector of inflammatory cytokine in the intestine during CD and UC, which facilitates the formation of inflammasomes in the bowel. However, IL-18 became necessary due to its functions in maintaining the protective effect of NLRP3 in the intestine, insofar as IL-18 deficiency was associated with decreased gut protection against exacerbated inflammatory responses. In this scenario, NLRP3 can translate danger and microbial sensors into robust immune responses via NF-kB and MAPK cascades. Among mucosal cells, NLRP3 instigates pyroptosis via an activating cleavage of gasdermin D (GSDMD), an executioner for pyroptosis [10,91].

Emerging evidence indicates that the persistent activation of the NLRP3 inflammasome is a crucial factor in the development of IBD [92,93,94]. As a result, targeting the NLRP3 inflammasome has been identified as a potential therapeutic approach for treating IBD. The presence of adenosine diphosphate, abundant in injured colonic tissue, has been found to activate the NLRP3 inflammasome by regulating P2Y1 receptor-mediated calcium signaling. This activation leads to the maturation and secretion of IL-1β, further exacerbating the progression of colitis [95,96,97].

Conversely, studies have demonstrated that the genetic ablation or pharmacological blockade of the P2Y1 receptor significantly alleviates DSS-induced colitis and endotoxic shock [95]. Moreover, it has been discovered that the Breast cancer type 1 susceptibility protein 1/2 (BRCA1/BRCA2)-containing complex 3 and Josephin domain containing 2 facilitate NLRP3-R779C deubiquitination and the interaction between serine/threonine-protein kinase NEK7 and NLRP3 [98,99,100,101]. These interactions promote NLRP3 inflammasome activation, thereby increasing the risk of developing IBD.

In IBD, monocytes, macrophages, and dendritic cells produce IL-1β. The leading producers are macrophages presented in the intestine lamina propria. Significantly, IL-1β triggers T cell proliferation during IBD and directly influences neutrophils recruitment to local inflammatory sites of the gut via combination with IL-1R and further activation of signaling cascades that culminate in NF-kB and MAPK pathways activation. Currently, NLRP3’s typical mediators start to divide holophotes with other pro-inflammatory cytokines such as TNF-α. Meanwhile, IL-1β also upregulates IL-2 receptor expression in T cells, which prolongs their survival and enhance their antibody production by direct B cell proliferation [10,102].

### 4.4. Medicinal Plants TARGETING NLRP3 inflammasome

#### 4.4.1. Xianglian Pill

To test the effects of the Xianglian Pill, a composition of *Coptis chinensis* Franch, *Evodia rutaecarpa*, and *Aucklandia lappa* Decne, against NLRP3 activation in IBD, Dai et al. [15] studied a DSS-induced C57BL/6 mice model of colitis. The results showed that after the treatment, the animals presented decreased levels of NLRP3, caspase-1, GSDMD-N, TLR4, *myeloid* differentiation protein (MyD88), NF-κB, p–NF–κB, IL-1β, TNF-α, IL-18, and *myeloperoxidase* (MPO).

#### 4.4.2. *Litsea cubeba*

Wong et al. [16] studied the effects of *Litsea cubeba* against NLRP3 activation in both an in vitro and in vivo models of IBD. After the treatment, the LPS and ATP stimulated J774A.1 cells presented decreased IL-1β, ASC, caspase-1, NLRP3, ROS, pyroptosis, IL-6, and mitochondrial ROS/damage relation. In addition, the DSS-induced C57BL/6 mice model showed decreased levels of the pro-inflammatory cytokines IL-1β and IL-6.

#### 4.4.3. *Artemisia anomala*

Hong et al. [17] evaluated the effects of *Artemisia anomala* against NLRP3 activation in IBD in vitro and in vivo models. After the treatment, the LPS-stimulated BMDMs cells presented decreased IL-1β, NLRP3, ASC, mitogen-activated protein kinase kinase kinase 7-c-jun n-terminal kinase (TAK1-JNK), caspase-1, p65 nuclear, IkappaB kinase-α (IκBα), NF-kB, lysosomal disruption, ROS, mitochondrial damage, and TNF-α expression levels. In addition, DSS-induced C57BL/6 mice model of colitis presented decreased levels of IL-1β.

#### 4.4.4. *Schisandra chinensis* (Turcz.) Baill

Bian et al. [18] utilized *Schisandra chinensis* (Turcz.) Baill against NLRP3 activation in a DSS-induced C57BL/6 mice model of colitis. The results showed that after the treatment the animals presented increased *superoxide dismutase* (SOD) and zonula occludens protein *1* (ZO-1) expression levels. In addition, the mice showed decreased expression levels of *malondialdehyde* (MDA), MPO, IL-1β, TNF-α, IL-18, TLR4, p-p65 and p-IκB-α, TLR4/NF-κB signaling, and NLRP3.

#### 4.4.5. Wu-Mei-Wan

Yan et al. [19] used Wu-Mei-Wan, a mixture of *Coptidis* rhizoma, *Phellodendri chinensis* cortex, *Zingiberis* rhizoma recens, *Typhonii* rhizoma, *Zanthoxyli*, *Mume* fructus, and *Ginseng radix* rhizome, against NLRP3 activation in a DSS-induced C57BL/6 mice model of colitis. The results showed that after the treatment, the animals presented decreased levels of NLRP3, Notch-1, NF-κBp65, p-NF-κBp65, NLRP3, IL-18, IL-6, IL-1β, co-expression of Notch-1, IL-18, TNF-α, macrophages infiltration, and interferon regulatory factor 5 (IRF5).

#### 4.4.6. Kui Jie Tong

Xue et al. [20] used Kui Jie Tong, a mixture of *Verbenae* herb, *Euphorbiae Humifusae* herb, *Arecae semen*, *Aurantii fructus* immaturus and *Angelicae sinensis* radix, against NLRP3 activation in a DSS-induced mice model of colitis. The results demonstrated that after the treatment, the animals presented NLRP3, ASC, caspase-1, never in mitosis gene a (NIMA) related kinase 7 (NEK7), pyroptosis, IL-1β, IL-18, IL-33, and GSDMD.

#### 4.4.7. *Piper nigrum*

In turn, Sudeep et al. [21] evaluated the effects of *Piper nigrum* against NLRP3 activation in a DSS-induced BALB/c mice model of colitis. The results demonstrated that the treated animals presented decreased TNF-α, IL-1β, NLRP3, oxidative stress (OS), and MDA, as well as increased claudin-1, occludin, SOD, catalase (CAT), and *glutathione* (GSH).

#### 4.4.8. *Morus macroura* Miq

Salama et al. [22] conducted a study with an AA-induced mice model of colitis to evaluate the effects of *Morus macroura* Miq against NLRP3 activation during this model of IBD. The results showed that, after treatment, the animals presented increased levels of micro ribonucleic acid 223 (miRNA-223), SOD and GSH, as well as decreased levels of TNF-α, NFκB-p65, caspase-1, NLRP3, MDA, IL-1β, TNF-α, IL-18, ROS, and nitrate/nitrite relation. 

#### 4.4.9. *Patrinia villosa*

Wang et al. [23] conducted an in vivo study with a TNBS-induced Sprague-Dawley rat model of colitis to evaluate the roles of *Patrinia villosa* against NLRP3 activation in this model of IBD. The results showed that the plant dry leaf extract could effectively decrease the expression of IL-1β, TNF-α, IL-6, NF-κB, p-NF-κB, NLRP3, and caspase-1 among the treated animals.

#### 4.4.10. *Ficus pandurata* Hance

Dai et al. [24] studied a DSS-induced C57BL/6 mice model of colitis to evaluate the roles of *Ficus pandurata* Hance against NLRP3 activation in this model of IBD. The results showed that the treatment with stem, leaf, root, and whole plant extracts could effectively decrease the animals’ expression of TLR4, MyD88, NF-κB, phospho-NF-κB, MDA, kelch-like ECH-associated protein 1 (Keap1), NADPH oxidase 2 (NOX2), and p22-phox, as well as increase their expression of Nrf2, heme oxygenase 1 (HO1), NAD(P)Hquinone-oxidoreductase-1(NQO1), total superoxide dismutase (T-SOD), and glutathione peroxidase (GSH-Px).

#### 4.4.11. *Agrimonia pilosa*

In turn, Li et al. [25] conducted an experiment with a DSS-induced C57BL/6 mice model of colitis to evaluate the effects of *Agrimonia pilosa* against NLRP3 activation. The results showed that during the IBD occurrence, the models presented decreased expression levels of TNF-α, IL-6, IL-1β, p65, p-p65, ASC, caspase-1, NF-kB, and NLRP3.

#### 4.4.12. *Vitis vinifera*

Sheng et al. [26] in an in vivo study with a DSS-induced C57BL/6 mice model of colitis, experimented to assess the roles of *Vitis vinifera* against NLRP3 activation in this model of IBD. The seed proanthocyanidin extract promoted decreases in the expression levels of TNF-α, IL-1β, NLRP3, ASC, caspase-1, and MDA. In addition, the results demonstrated an increased expression of IL-10, SOD, and GSH.

#### 4.4.13. *Lycium ruthenicum* Murray

Zong et al. [27] evaluated the roles of *Lycium ruthenicum* Murray against NLRP3 activation in a DSS-induced C57BL/6 mice model of colitis. The results demonstrated that the treated animals presented decreased expression levels of TNF-α, IL-1β, IL-6, IL-17A, IFN-γ, TLR4, p-IκB, NF-κB, p65, p-p65, c-Jun, p-signaling transducer and activator of transcription 3 (STAT3), cyclooxygenase-2 (COX-2), induced nitric oxide synthase (iNOS), nitric oxide (NO), *prostaglandin E2* (PGE2), p38 phosphorylation, ERK phosphorylation, c-Jun N-terminal kinase (JNK) phosphorylation, NLRP3, ASC, caspase-1, IL-1β, ROS, and MDA, as well as increased expression levels of IL-10, CAT, SOD, and GSH.

### 4.5. Phytochemicals That Target NLRP3 Inflammasome

#### 4.5.1. Artemisitene

In an experimental study with in vitro and in vivo models of colitis, Hua et al. [28] used artemisitene, a derivative of artemisinin from *Artemisia annua* plant, against NLRP3 activation in IBD. The results showed that with the treatment, the LPS, nigericin, or ATP-stimulated J774A.1 cells and LPS, nigericin, NLR family CARD domain containing 4 (NLRC4), and absent in melanoma 2 (AIM2)-stimulated BMDMs cells presented decreased IL-1β, NLRP3-mediated IL-1β secretion, NF-κB-dependent TNF-α activation, pro-caspase-1 cleavage, the interaction between NLRP3 and ASC, ASC oligomerization, ASC specks, NLRP3-ASC binding, NLRC4, AIM2, and IL-6. In addition, the DSS-induced C57BL/6 mice showed decreased IL-1β, TNF-α, and IL-6 serum levels.

#### 4.5.2. Morroniside

S. Zhang et al. [29] studied LPS-stimulated NCM460 cells in vitro to evaluate the effects of morroniside, an iridoid glycoside derived from *Cornus offinalis*, against NLRP3 activation during this model of IBD. The results showed that with the treatment, the cells presented regulation of the NLRP3 activation through decreased BCL-2-associated X-protein (BAX), TNF-α, IL-1β and IL-6, MDA, and MPO expressions, NLRP3 signaling and p-p65–p65 and p-IκBα–IκBα expressions, as well as increased B-cell lymphoma protein 2 (BCL-2), SOD, and total antioxidant capacity (T-AOC) expressions.

#### 4.5.3. Protopine

In an in vitro study with LPS-stimulated NCM460 cells, J. Li et al. [30] evaluated the roles of protopine, a derivative of the poppy, berberi, walnut, and buttercup families, against this model of IBD. The results showed that, with the treatment, the cells controlled NLRP3 activation through decreased BAX, TNF-α, IL-1β, IL-6, MDA, MPO, ROS, intracellular Ca^2+^ concentration, NLRP3 signaling, p-IκBα/IκBα, and p-P65/P65, as well as increased BCL-2, T-AOC, SOD, and mitochondrial membrane potential. 

#### 4.5.4. Ferulic Acid

Yu et al. [31] proposed ferulic acid, a phenolic compound derived from diverse fruits and vegetables, as an effective treatment against NLRP3 activation in IBD both in vitro and in vivo. The results showed that with the treatment, the TNF-α-stimulated HIMECs cells presented decreased IL-1β, IL-6, IL-12, caspase-1, caspase-3, BCL-2, and thioredoxin-interacting protein (TXNIP)/NLRP3 levels. In addition, the TNBS-induced Sprague-Dawley mice model of colitis presented decreased IL-1β, IL-6, IL-12, caspase-1, caspase-3, TXNIP, and NLRP3 levels.

#### 4.5.5. Artemisinin Analog SM934

Shao et al. [32] innovated using the artemisinin analog SM934, which is water soluble and isolated from *Artemisia annua*, as an effective treatment against NLRP3 signaling activation in IBD both in vitro and in vivo. After treatment, the TNF-α-stimulated Caco-2 and HT-29 cells presented decreased c-Casp3, NLRP3, claudin-2, ASC, c-Casp1, IL-18, p-NF-κB, p-p38, p-ERK, p-JNK, GSDMD, GSDMD-F, and GSDMD-N, as well as increased E-cadherin, ZO-1, and occludin. In addition, the TNBS-induced C57BL/6 mice model of colitis presented decreased c-Casp3, BAX/BCL-2, c-Casp9, NLRP3, ASC, c-Casp1, GSDMD, IL-18, high mobility group box 1 (HMGB1), NF-κB, ERK, p38, and JNK.

#### 4.5.6. Betaine

Chen et al. [33] evaluated the roles of betaine, a bioactive compound richly found in sugar beet, against NLRP3 activation during the DSS-induced C57BL/6J mice model of colitis. After the treatment, the animals presented increased occludin, ZO-1, Nrf2, CAT, and SOD expression levels and decreased MDA, MPO, NOS-related enzymes, COX2, and NLRP3, respectively, ASC, c-Casp1, and N-terminal GSDMD.

#### 4.5.7. N-Palmitoyl-D-glucosamine

In an in vivo study with dinitrobenzene sulfonic acid (DNBS)-induced C57BL/6J mice model of colitis, Palenca et al. [34] evaluated the roles of N-palmitoyl-D-glucosamine, a natural amide of palmitic acid and glucosamine that belongs to the ALIAmide (autacoid local injury antagonist) family, against this model of IBD. As a result, the animals presented elevated occludin and ZO-1 expression levels and decreased TLR-4, NLRP3, iNOS, IL-1β, and PGE2 expression levels.

#### 4.5.8. Moronic Acid

In an in vitro and in vivo study using IFN-γ-stimulated intestinal macrophages and DSS-induced C57BL/6 mice model of colitis, respectively, Ruan and Zha [35] assessed the roles of moronic acid against a triterpenoid richly found in *Pistacia chinensis*, against NLRP3 activation during IBD. The results showed that with treatment, the cells expressed decreased TNF-α, IL-1β, IL-6, ROS, CD11, NF-kB (P50), NLRP3, p-P50, and M1 macrophage polarization. In addition, the animals presented with treatment-decreased levels of ROS, CD11, TNF-α, IL-1β, IL-6, ZO-1, NLRP3, and p-P50.

#### 4.5.9. Munronoid I

Ma et al. [36] studied LPS/ATP-stimulated mouse peritoneal macrophages and BMDMs cells in vitro and DSS-induced C57BL/6 mice colitis model in vivo to evaluate the effects of munronoid I, a diterpenoid richly extracted from the *Meliaceae* family, against NLRP3 activation during IBD. The treated cells presented decreased expression levels of caspase-1 p20, IL-1β, IL-18, NLRP3, and GSDMD p30. In addition, the treated animals showed reduced expression levels of NLRP3, cleaved caspase-1 (p20), pyroptosis-related protein cleaved GSDMD (p30), IL-6, TNF-α, IL-1β, and IL-18.

#### 4.5.10. Sanguinarine

Studying LPS-induced THP-1 cells in vitro and DSS-induced C57BL/6 mice model of colitis in vivo, X. Li et al. [37] assessed the effects of sanguinarine, a benzophenanthridine that belongs to the group of benzylisoquinoline alkaloids and is extracted from *Argemone mexicana* L., *Chelidonium majus* L., *Macleaya cordata* (Willd.) R. Br., *Sanguinaria canadensis* L. and *Bocconia frutescens* L., against NLRP3 activation during IBD. The results showed that the treated cells presented decreased expression levels of NLRP3, caspase-1, IL-1β, ROS, and IL-18. In addition, the treated animals presented decreased expression levels of NLRP3, caspase-1, TNF-α, IFN-γ, IL-1β, IL-6, IL-13, and IL-18, as well as increased levels of IL-4 and IL-10.

#### 4.5.11. 8-Oxypalmatine

Cheng et al. [38] studied the effects of 8-oxypalmatine, an isoquinoline alkaloid derived from *Fibraureae caulis*, in a DSS-induced ulcerative colitis mice model. After the intervention, a reversal of the pattern of inflammatory mediators’ expression and secretion was observed, with a decrease in pro-inflammatory cytokines such as TNF-α, IL-1β, IFN-γ, IL-17, and IL-6. There was also an increase in the expression and secretion of the anti-inflammatory IL-10 and antioxidant enzymes such as T-AOC, SOD, GSH, CAT, and GSH-Px. At least, there was decreased messenger ribonucleic acid (mRNA) expression of NLRP3, ASC, and Caspase-1.

#### 4.5.12. Quercetin

H. X. Zhang et al. [39] evaluated the roles of quercetin, a bioactive compound richly found in many plants’ flowers, leaves, and fruits, against NLRP3 activation in IBD. The results showed that among treated LPS-induced rat intestinal microvascular endothelial cells (RIMVECs) in vitro, quercetin effectively reduced their expression of TLR4, NLRP3, caspase-1, GSDMD, IL-1β, IL-18, IL-6, and TNF-α.

#### 4.5.13. Picroside II

Yao et al. [40] conducted a study that evaluated the effects of picroside II, a flavonoid compound extracted from the traditional Chinese medicinal herb *Picrorhiza scrophulariiflora*, in DSS-induced colitis in C57BL/6 mice and LPS-stimulated THP-1 cells. The treatment showed that picroside II could decrease the expression and secretion levels of pro-inflammatory cytokines such as TNF-a, IL-6, and IL-1β both in vivo and in vitro, and reduce the activity of the NLRP3 inflammasome and the activation of the NF-kB.

#### 4.5.14. Hydroxytyrosol

Miao et al. [41] evaluated the role of hydroxytyrosol, a phenolic compound extracted from olive oil and olive leaves, in a mice model of DSS-induced colitis. After treatment, the models showed improvement in the inflammatory and oxidative profiles, modulation of the intestinal microbiota, and less activation of the NLRP3 inflammasome, measured by decreased mRNA transcription of NLRP3 inflammasome proteins. 

#### 4.5.15. SCLP

Pan et al. [42] studied the benefits of SCLP, a pectic polysaccharide purified from *Smilax china* L., on DSS-induced UC in vivo. With the treatment, there was a decrease in the inflammatory activity, with a decline in the expression and secretion of IL-6, TNF-α, IL-1β, MPO, Gal-3 protein and, consequently, lower expression of the NLRP3, ASC, and Caspase-1 proteins and, therefore, lower activation of the NLRP3 inflammasome.

#### 4.5.16. Dioscin

Cai et al. [44] conducted an in vivo study to observe the effects of dioscin, a natural steroidal saponin derived from plants of the genus *Dioscoreaceae*, on DSS-induced UC in rats. Treatment with dioscin promoted a change in the pattern of macrophages to M2, with consequent improvement in the pro-inflammatory cytokines’ profile. There was a reduction in the expression and secretion of TNF-α and IL-1β pro-inflammatory mediators and an increase in the IL-10. Dioscin also suppressed the activation of the NLRP3 inflammasome and the expression of MAPKp38 and NF-κBp65 proteins.

#### 4.5.17. Bryodulcosigenin

Li et al. [43] evaluated the potential of bryodulcosigenin, a cucurbitacin-type triterpenoid isolated from *Bryonia* roots, both in vitro and in vivo. The authors used TNF-α-stimulated NCM460 cells and a DSS-induced C57BL/6 mice model of colitis. After treatment, the models showed a decrease in the activity of the NLRP3 inflammasome with a consequent decrease in the expression and secretion of pro-inflammatory cytokines and apoptosis-related proteins and an increase in the expression of the epithelial occludin and ZO-1.

#### 4.5.18. Rosmarinic Acid

Marinho et al. [45] conducted an in vivo study to evaluate the action of rosmarinic acid, a natural polyphenol found in the *Labiatae* family of herbs, in DSS-induced UC in C57BL/6 mice. The treatment showed that rosmarinic acid decreased the expression and release of pro-inflammatory cytokines such as TNF-α and IL-1β, decreased MPO activity, promoted activation of the Nrf2 antioxidant pathways, and decreased the expression of NLRP3-related inflammasome proteins.

#### 4.5.19. Mogrol

Liang et al. [46] studied the activity of mogrol, an active component of Luo Han Guo, in DSS-induced colitis in C57BL/6 mice, phorbol 12-myristate 13-acetate (PMA)-stimulated THP-1 cells, and LPS-stimulated THP-M cells. Mogrol treatment decreased the expression and secretion of IL-1β and IL-17 pro-inflammatory cytokines, increased IL-10, occludin, and ZO-1, and decreased NLRP3 inflammasome activation and IκBα degradation in vivo. Additionally, there was increased occludin and ZO-1 in vitro, increased expression of BCL-2/BAX, adenosine monophosphate-activated *protein* kinase (AMPK) phosphorylation, and suppressed caspase-1 activation.

#### 4.5.20. Sinapic Acid

Qjan et al. [47] evaluated the role of sinapic acid, a naturally occurring hydroxycinnamic acid widely found in vegetables, fruits, cereals, and nuts, in a DSS-induced model of UC. The results showed that the treatment reduced the expression and release of the pro-inflammatory cytokines TNF-α, IL-1β, IL-6, IL-8, IL-17α, and IFN-γ while increasing the levels of the anti-inflammatory cytokines IL-4 and IL-10. The treatment also elevated the model’s antioxidant activity by elevating SOD, GSH-Px, CAT, and GSH antioxidant enzymes and reducing MDA. Sinapic acid also promoted an increase in claudin-1, occludin, and ZO-1 proteins and, finally, decreased the activity of the NLRP3 inflammasome with a decrease in NLRP3, ASC, and caspase-1 proteins expression.

#### 4.5.21. Evodiamine

Ding et al. [48] studied human THP-1 cells stimulated by LPS in vitro and DSS-induced C57BL/6J mice model of colitis in vivo to evaluate the effects of evodiamine, a bioactive compound richly found in the fruits of *Evodiae fructus*, against NLRP3 activation during IBD. After treatment, the cells presented decreased expression levels of IL-1β, IL-18, caspase1, ASC, ASC oligomers, and P62, and the animals presented decreased expression levels of MPO, IL-1β, IL-18, caspase1, ASC, p-P65NFκB, p-IκB, and P62. After treatment, both in vitro and in vivo models expressed increased levels of microtubule-associated protein light chain 3 (LC3), as well as decreased levels of NLRP3 inflammasome assembly.

#### 4.5.22. Geniposide

Pu et al. [49] studied geniposide, the main active ingredient extracted from *Gardenia jasminoides*, against NLRP3 activation during the following LPS-stimulated BMDM cells and RAW264.7 macrophages in vitro and DSS-induced C57BL/6J mice in vivo models of IBD. After treatment, the cells presented decreased expression levels of IL-1β and caspase-1, and the animals presented decreased expression levels of MPO, IL-1β, IL-17, TNF-α, IFN-γ, caspase-1, nitric oxide synthase 2 (NOS2) mRNA, and Arg1 mRNA. In addition, both in vitro and in vivo models presented reduced NLRP3 inflammasome pathway activation.

#### 4.5.23. Chlorogenic Acid

Zeng et al. [50] studied LPS/ATP-induced RAW264.7 macrophages in vitro and DSS-induced BALB/c mice colitis model in vivo to evaluate the roles of chlorogenic acid, an active dietary polyphenol richly found in honeysuckle, *Eucommia* and *Chaenomeles Lagenaria*, against NLRP3 activation during IBD. The results showed that after treatment, both in vitro and in vivo models presented decreased expression levels of IL-1β, IL-18, NLRP3, ASC, caspase1 p45, caspase1 p20, NF-kB, and micro ribonucleic acid 155 (MiR-155).

#### 4.5.24. Resveratrol

Sun et al. [51] studied the protective effect of resveratrol, a natural non-flavonoid polyphenol significantly present in grapes and wine, against radiation-induced IBD via NLRP-3 inflammasome repression in C57/6 mice. After treatment, the animals presented decreased NLRP3, Sirt1, IL-1β, and TNF-α expression levels.

#### 4.5.25. Ginsenoside Rk3

Tian et al. [52] studied the effects of ginsenoside Rk3, a bioactive compound richly found in ginseng, against NLRP3 activation during a DSS-induced C57BL/6 mice model colitis. The results showed that after treatment, the animals presented decreased NLRP3, ASC, caspase1, MPO, iNOS, IL-1β, TNF-α, and IL-6 expression levels and increased claudin 1, occludin, and ZO-1.

#### 4.5.26. Physalin B

Zhang et al. [53] studied physalin B, a main active withanolide in *Physalis alkekengi* L. var. franchetii (Mast.) Makino, against NLRP3 during LPS-stimulated RAW 264.7 macrophages in vitro and DSS-induced BALB/c mice model of acute colitis in vivo models of IBD. The results showed that, after treatment, the cells presented decreased IL-1β, IL-6, and TNF-α expression levels, and the animals showed decreased NLRP3, ASC, IL-1β, MPO, TNF-α, IL-6, NF-κB activation cascade and STAT3 expression levels and β-arrestin one signaling pathway activation.

#### 4.5.27. Oroxindin

Liu et al. [54] studied the effects of oroxindin, a flavonoid isolated from the traditional Chinese medicine Huang-Qin, against NLRP3 activation during LPS-stimulated human THP-1 cells in vitro and DSS-induced C57BL/6 mice model of acute colitis in vivo models of IBD. The results showed that after treatment, the cells and animals presented decreased levels of NLRP3, IL-1β, caspase1, and IL-18.

#### 4.5.28. Resveratrol Analog 2-Methoxyl-3,6-dihydroxyl-IRA

Chen et al. [55] studied the effects of the synthetic imine Resveratrol Analog 2-Methoxyl-3,6-Dihydroxyl-IRA against NLRP3 activation during human colon cancer LS174T and Caco2 cells in vitro and DSS-induced C57BL/6 mice model of colitis in vivo models of IBD. The results showed that after treatment, both in vitro and in vivo models presented decreased levels of NLRP3, TNF-α, and IL-6 expression levels and increased Nrf2, aldo-keto reductase family 1 member C (AKR1C), NQO1, and GSH.

#### 4.5.29. Curcumin

**Figure 4:**
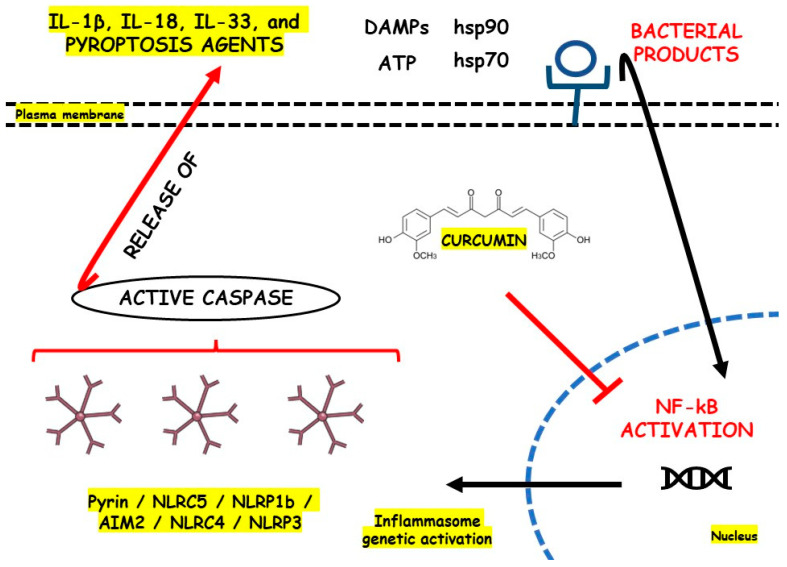
NLRP3 inflammasome regulation with the use of curcumin. AIM2, Interferon-Inducible Protein AIM2; ATP, Adenosine Triphosphate; DAMPs, Damage-Associated Molecular Patterns; hsp70, Heat Shock Protein 70; hsp90, Heat Shock Protein 90; IL, Interleukin; NF-kB, Nuclear Factor Kappa b; NLR, Nod-like Receptor; NLRC5, NOD-like receptor family CARD domain containing 4; NLRC5, NOD-like receptor family CARD domain containing 5; NLRP1b, NLR family pyrin domain containing 1b; NLRP3, Nod-like Receptor Family Pyrin Domain Containing 3.

Gong et al. [56] studied LPS-primed peritoneal macrophages in vitro and DSS-induced C57BL/6 mice model of colitis in vivo to evaluate the roles of curcumin, a bioactive phenolic compound richly found in *Curcuma longa*, against NLRP3 activation during these models of IBD. The results showed that after treatment, the cells presented decreased levels of IL-1β, ROS production, and cathepsin B leakage and increased levels of K^+^ efflux. In turn, after treatment, the animals presented decreased NLRP3, IL-1β, ASC, ROS, cathepsin B leakage, caspase1, MPO, monocyte chemoattractant protein-1 (MCP-1), and IL-6 levels and increased K^+^ efflux. Figure 4 illustrates the NLRP3 regulation with the use of curcumin.

## 5. Future Research Directions

In summary, CD and UC are prevalent diseases associated primarily with a massive inflammatory response derived from NLRP3 inflammasome domain activation. Even during their first stages, IBD progression highly depends on activating inflammatory pathways. In recent years, the clinical goal of regulating NLRP3 during these conditions became a field of study. Although synthetic anti-inflammatory and immunomodulatory drugs are necessary to treat IBD, they can lead to several adverse effects. Anti-inflammatory natural compounds exert minimal undesirable effects. Consequently, there is a growing interest in exploring phytochemicals and medicinal plants as a source of innovative treatments to inhibit NLRP3 activation and address the pathophysiology of CD and UC. The modulatory mechanisms involved in NLRP3 regulation by phytochemicals and medicinal plants result mainly in decreased levels of TNF-α, IL-1β, caspase, ASC, MDA, IL-18, IL-6, p-NF-kB, TLR4, NOX2, and ↓p22-phox, as well as increased levels of Nrf2, HO1, NQO1, IL-10, IL-4, SOD, GSH, ZO-1, claudin 1, and occludin. These effects can bring several benefits to IBD therapeutic approaches.

Although medicinal plants and their primary phytochemicals are promising therapeutics in treating intestinal diseases such as UC and CD, they often have low oral absorption rates and limited biological distribution through the human systems. Therefore, their systemic bioavailability still restricts their clinical application [2,6,13]. Nanotechnology’s development using nanocarriers has recently opened new possibilities for treating IBD with nanoparticle-based drug approaches. These delivery systems attract particular attention due to their small size, low immunogenicity, surface modification diversity, and targeting advantages [103]. At present, there is a minimal number of clinical trials that have been conducted using medicinal plants and their phytochemicals as NRLP3 modulators. NLRP3 inflammasome indeed exerts deleterious effects during IBD pathogenesis. In turn, reintegrating the potential shown in this manuscript of medicinal herbs and their phytocompounds in treating inflammatory bowel-related conditions, it is crucial to conduct further experiments and clinical trials focusing on IBD therapy through NLRP3-modulating natural agents. Shortly, numerous clinical studies will be necessary to validate the therapeutic benefits of phytochemicals that target NLRP3 in treating IBD due to the elevated prices of synthetic medicines and their deleterious adverse events [104,105,106]. In the current conditions of global environmental change and also significant declines in fauna and flora biodiversity, preserving medicinal plants for treating human illnesses and maintaining the associated local ecological knowledge plays a fundamental role in not only achieving the Sustainable Development Goal of promoting good health but also well-being for all [2,107,108,109].
